# Evaluating Binary Molybdenum Alloys as Strong and Ductile High-Temperature Materials

**DOI:** 10.3390/ma18143329

**Published:** 2025-07-15

**Authors:** Cheng Fu, Jiayi Yan, Jiang Yu, Yuhong Ren, Sha Li

**Affiliations:** 1 CNNC Jianzhong Nuclear Fuel Co., Ltd., Yibin 644000, China; 2 Key Laboratory of Advanced Materials (MOE), School of Materials Science and Engineering, Tsinghua University, Beijing 100084, China

**Keywords:** molybdenum alloys, thermodynamics, modeling, mechanical properties, composition optimization

## Abstract

Molybdenum alloys as refractory alloys can provide strength levels at operating temperatures higher than that of Ni-base superalloys, yet their ductility is usually inferior to Ni-base alloys. Currently, commercialized Mo alloys are much fewer than Ni alloys. The motivation of this work is to explore opportunities of discovering useful alloys from the usually less investigated binary Mo-X systems (X = alloying element). With computational thermodynamics (CALPHAD), first-principles calculation, and mechanistic modeling combined, in this work a large number of Mo-X binary systems are investigated in terms of thermodynamic features and mechanical properties (yield strength, ductility, ductile-brittle transition temperature, creep resistance, and stress-strain relationship). The applicability of the alloy systems as solution-strengthened or precipitation-strengthened alloys is investigated. Starting from 92 Mo-X systems, a down-selection process is implemented, the results of which include three candidate systems for precipitation strengthening (Mo-B, Mo-C, Mo-Si) and one system (Mo-Re) for solid-solution strengthened alloy. In a composition optimization of Mo alloys to reach the properties of Ni-base superalloys, improving ductility is of top priority, for which Re plays a unique role. The presented workflow is also applicable to other bcc refractory alloy systems.

## 1. Introduction

Molybdenum is a rare refractory metal with a melting point as high as 2620 °C, making it second only to carbon, tungsten, rhenium, tantalum, and niobium in density. Molybdenum exhibits excellent thermal and electrical conductivity, high strength, outstanding creep resistance, a low coefficient of thermal expansion, and strong corrosion resistance [1,2,3]. It is widely used as a high-temperature functional and structural material in aerospace, aviation, and nuclear industries, and is considered one of the refractory metals with significant application potential [4]. However, issues inherent to the structure of molybdenum, such as low-temperature brittleness and insufficient high-temperature oxidation resistance, limit its widespread use and extensive processing. Alloying is an effective approach to improve these properties [5,6,7]. In fact, alloying is the primary means to enhance the performance of molybdenum, and high-temperature Mo alloys demonstrate superior mechanical properties compared to pure molybdenum. For example, up to around 1700 °C, the yield strength and tensile strength of Mo alloys are far higher than those of pure Mo. Even at 1800 °C, high-temperature Mo alloys retain substantial elongation and excellent creep performance [8]. Some recent advances related to finding strong and ductile Mo alloys include the following. The Mo-1.0B alloy exhibits an ultimate tensile strength of 1016.6 MPa and a total elongation of 8.6% at room temperature, which is superior to pure molybdenum’s 916.3 MPa and 6.5%. After annealing at 1200 °C, Mo-1.0B elongation is 67.4%, which is 43.7% higher than pure molybdenum [9]; a cerium partially stabilized zirconia (Ce-PSZ) reinforced molybdenum alloy with outstanding properties was obtained, and when 1.5 wt% of Ce-PSZ is added, the Mo alloys achieve excellent overall mechanical properties, with an ultimate tensile strength of 528.5 MPa, elongation of 29.3%, and fracture toughness of 38.5 MPa∙m^1/2^. These values are about 22.7%, 22.5%, and 83.3% higher than those of pure molybdenum [10]; solid-solution microalloying of Mo—via additions of W, Re, Nb, Ti or Zr—induces lattice distortions that impede dislocation motion and thereby markedly enhance both strength and high-temperature creep resistance relative to pure Mo. In particular, W and Re solutes elevate yield strength and thermal stability, while trace Ti or Zr refine grains and raise hardness [11].

Molybdenum alloys are categorized as solution-strengthened, particle-strengthened, dispersion-strengthened, or with some other less common strengthening mechanisms. Therefore, further investigation of the mechanisms by which strength and toughness are enhanced in Mo alloys, and clarification of how different alloying elements affect the room-temperature and high-temperature mechanical properties of Mo alloys, can effectively advance the development of production and processing techniques for novel high-performance Mo alloys.

Currently, the development of Mo alloys is relatively lagging; compared with traditional structural materials like steels and Ni-based alloys, there are very few Mo alloy grades and they are relatively simple. More importantly, the selection of alloying elements and the systematic, scientific evaluation of their alloying effects are lacking, and progress still depends heavily on empirical data and limited experimental results. This situation, to some extent, hinders the broader application and further development of Mo alloys in certain high-temperature non-aqueous environments. However, molybdenum alloy design can be accelerated by modern computational techniques, such as computational thermodynamics and kinetics (CALPHAD, CALculation of PHAse Diagrams) [12] and density functional theory (DFT) [13] calculations. It is usually more convenient to investigate a large number of alloy systems computationally than experimentally. By applying some design criteria and constraints to the computational results, candidate alloys fulfilling the design requirements can be determined and considered for experimental validation, reducing the cost and time of experimental trial-and-error. This study is a comprehensive investigation of 92 molybdenum-containing binary alloy systems (Mo-X). The motivation is to explore the opportunity of finding useful alloys from the usually less investigated binary molybdenum alloy systems. The purpose of this work is to look for new directions of alloying for good mechanical properties based on solid-solution strengthening and precipitation strengthening mechanisms. Thermodynamic calculation, first-principles calculation, mathematical modeling, and literature survey are combined to investigate binary molybdenum alloy systems, In this work, we apply a combination of CALPHAD calculations, DFT calculations, modeling, and literature survey, to categorize a large number of binary alloy systems and down-select them to a small number of alloy systems suitable for solution-strengthening and precipitation-strengthening mechanisms. During the down-selecting process, modeling of strength, ductility, ductile-to-brittle transition temperature (DBTT), creep, and stress-strain behavior are implemented. Combining the results of modeling and experimental data from the literature, we propose some future directions of Mo alloy development.

## 2. Methods

The methods used in this work include thermodynamic calculations, density functional theory calculations, mathematical modeling (including curve fitting), literature survey, etc. In this section we elaborate on the methods which contains some more details.

### 2.1. Thermodynamic Calculation

Thermodynamic calculations in this work are performed using the software Thermo-Calc [14] version 2023a, and with TCNI10 thermodynamic database and MOBNI5 mobility database, unless specified otherwise. For prescribed temperature and alloy composition, standard equilibrium calculations give results including stable phases and their phase fraction, composition, sublattice site occupancy, activity and chemical potential of components, and many more. Mapping over the composition-temperature plane, Thermo-Calc can calculate and present binary equilibrium phase diagrams. The driving force of a precipitate phase from a supersaturated solid solution can also be calculated, with the precipitate phase set to “dormant” status and the matrix phase set to “entered”, representing the initial status of precipitation.

### 2.2. DFT Calculation

DFT calculations are performed to investigate atomic-scale structures and energetics of solid solutions, using the software Vienna Ab initio Simulation Package (VASP) [15,16,17] version 6.4.2, with the projector-augmented-wave (PAW) method [18,19] and the PBEsol exchange-correlation functional [20]. Plane-wave energy cutoff is set to 500 eV and the k-point mesh is 4 × 4 × 4 of the Monkhorst-Pack [21] type.

BCC supercells are generated and optimized towards ideally random configurations using a Monte Carlo method implemented in the code spcm. The supercells used in this work for binary Mo-X alloys containing 4 × 4 × 4 unit cells, i.e., 128 atoms, having the compositions Mo_1-c_X_c_ with c = 0.125, 0.25, 0.375, 0.5. After the optimization, the Warren-Cowley short-range order parameters in the first eight coordinate shells are less than 0.05 in magnitude, meaning the supercells represent random solid solutions well. The equilibrium structures of these binary-alloy supercells are obtained by relaxing internal positions of atoms while keeping the supercell shape cubic. The relaxation finishes until all force components of all atoms are less than 0.01 eV/Å.

## 3. Thermodynamic Characteristics of Binary Molybdenum Alloys

The 92 alloying elements under consideration include: Ac, Ag, Al, Am, As, Au, B, Ba, Be, Bi, Bk, Br, C, Ca, Cd, Ce, Cl, Cm, Co, Cr, Cs, Cu, Dy, Er, Es, Eu, F, Fe, Fm, Fr, Ga, Gd, Ge, H, Hf, Hg, Ho, I, In, Ir, K, La, Li, Lr, Lu, Mg, Mn, N, Na, Nb, Nd, Ni, No, Np, O, Os, P, Pa, Pb, Pd, Pm, Pr, Pt, Pu, Ra, Rb, Re, Rh, Ru, S, Sb, Sc, Se, Si, Sm, Sn, Sr, Ta, Tb, Tc, Te, Th, Ti, Tl, Tm, U, V, W, Y, Yb, Zn, and Zr. Since the focus of this research is on structural material applications, 17 radioactive elements (Tc, Pm, Fr, Ra, Ac, Th, Pa, U, Np, Pu, Am, Cm, Bk, Es, Fm, No, and Lr) are excluded from further consideration. We first investigate the thermodynamic features of the remaining 75 Mo-X systems, which determine the possibility of realizing solid-solution strengthening or precipitation strengthening.

### 3.1. Binary Phase Diagrams

Binary Mo-X phase diagrams give some fundamental and general features of the alloy systems. Ways to obtain binary phase diagrams include (in order):

- Thermo-Calc calculation using TCNI10 and (for systems not assessed in TCNI10) other Thermo-Calc databases;
- Thermo-Calc calculation using thermodynamic assessment in the literature;
- Handbooks of binary phase diagrams.

The sources of phase diagrams used in this work are listed below in Table 1. The Mo-X systems corresponding to the TC-databases are assessed in the full range of composition and temperature, according to the technical information sheets of the TC-databases.

Based on features of the binary equilibrium phase diagrams, binary Mo alloys can be categorized according to solubility of element X in the bcc-Mo matrix and the presence of secondary phases. The categories are as follows.

#### 3.1.1. Type I: Continuous bcc-Mo-X Solid Solution

**Figure 1:**
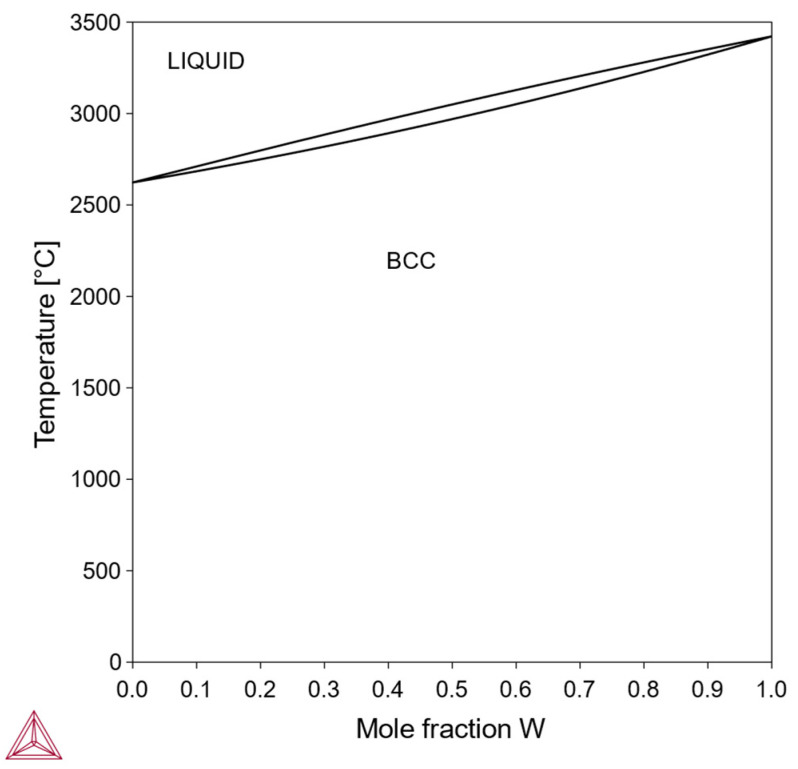
Mo-W phase diagram, with a continuous bcc solid solution (Thermo-Calc).

In this type, the pure element X can also exist stably in a bcc structure, and the Mo-X phase diagram contains a single-phase bcc region across the entire composition range. An example is the Mo-W system (Figure 1). Alloys of this type can dissolve a high concentration of the alloying element and are suitable for designing solid-solution-strengthened Mo alloys.

#### 3.1.2. Type II: Limited Solubility with Intermediate Phases

**Figure 2:**
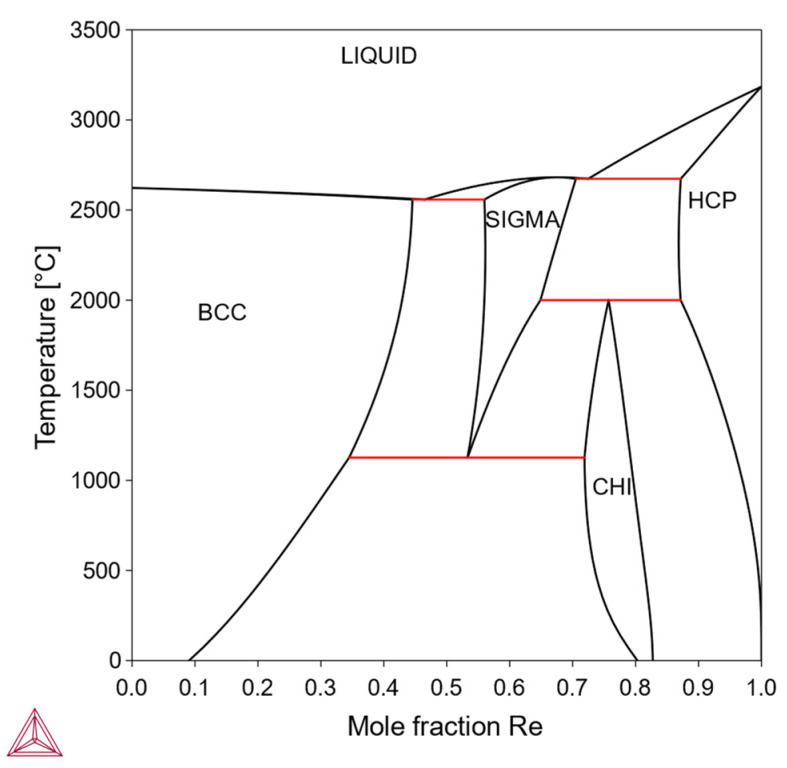
Mo-Re phase diagram. The solubility of Re in bcc solid solution being limited by sigma and chi phases (Thermo-Calc).

**Figure 3:**
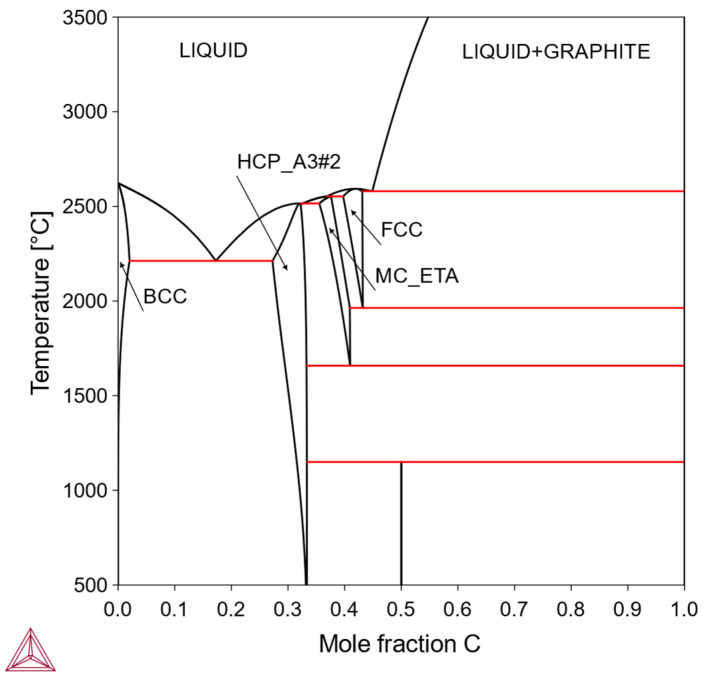
Mo-C phase diagram. The solubility of C in bcc phase is limited by Mo_2_C (HCP_A3#2) phase (Thermo-Calc).

In this type, the solubility of X in the bcc-Mo matrix is finite, being limited by the formation of one or more intermediate phases. Within the solubility limit, it is possible to make solid-solution-strengthened alloys. Otherwise, it is also possible to make precipitation-strengthened alloys via a solutionizing-aging treatment. Solutionizing treatment is performed in a single-phase region while the aging treatment is performed in a two-phase region; therefore, the solubility curve on the phase diagram is an important feature for designing alloy composition and heat treatment. Examples include the Mo-Re system (Figure 2) and the Mo-C system (Figure 3).

#### 3.1.3. Type III: Limited Solubility Without Intermediate Phases

In this type, X is also to some extend soluble in the bcc-Mo matrix, but the solubility is now limited by the substance of pure X. Since pure substance phases are usually too soft to act as strengthening precipitates, in this type only solid-solution strengthening is possible. An example is the Mo-Lu alloy system [27].

#### 3.1.4. Type IV: Negligible Solubility in bcc Mo, Intermediate Phase Present

**Figure 4:**
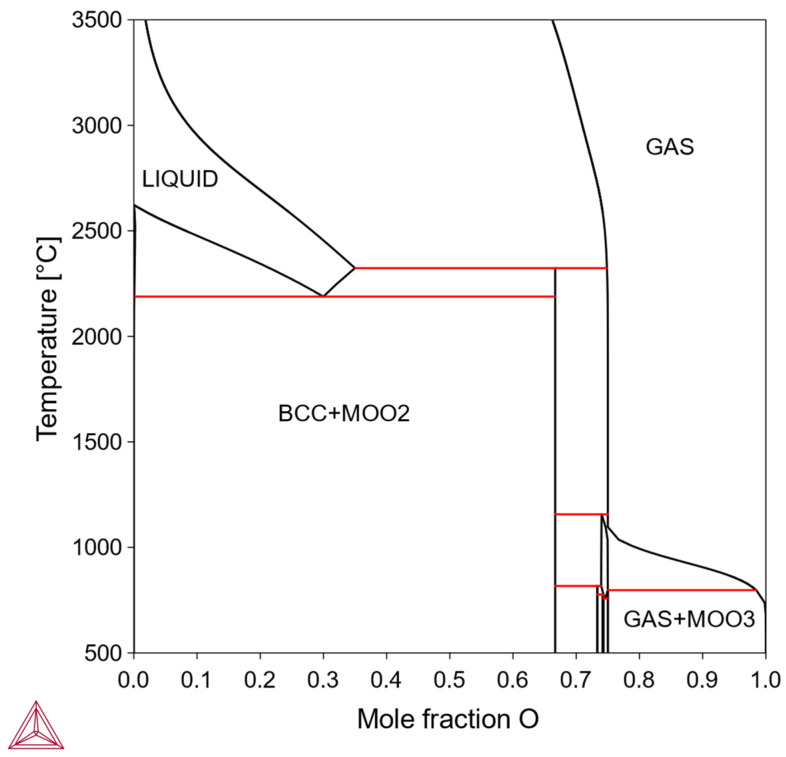
Mo-O phase diagram (Thermo-Calc).

In this type, the solubility of X is so limited that practical solution-aging treatments are difficult to achieve, making solid-solution strengthening or precipitation strengthening impractical. In theory, if the alloy is directly prepared in the two-phase region and sintered, dispersion strengthening might be possible. But the feasibility depends on the mechanical properties of the intermediate phase, its interface with the bcc Mo matrix, and some other factors about processibility. Examples of such binary alloys include the Mo-O system (Figure 4).

#### 3.1.5. Type V: Negligible Solubility in bcc Mo, No Intermediate Phase

**Figure 5:**
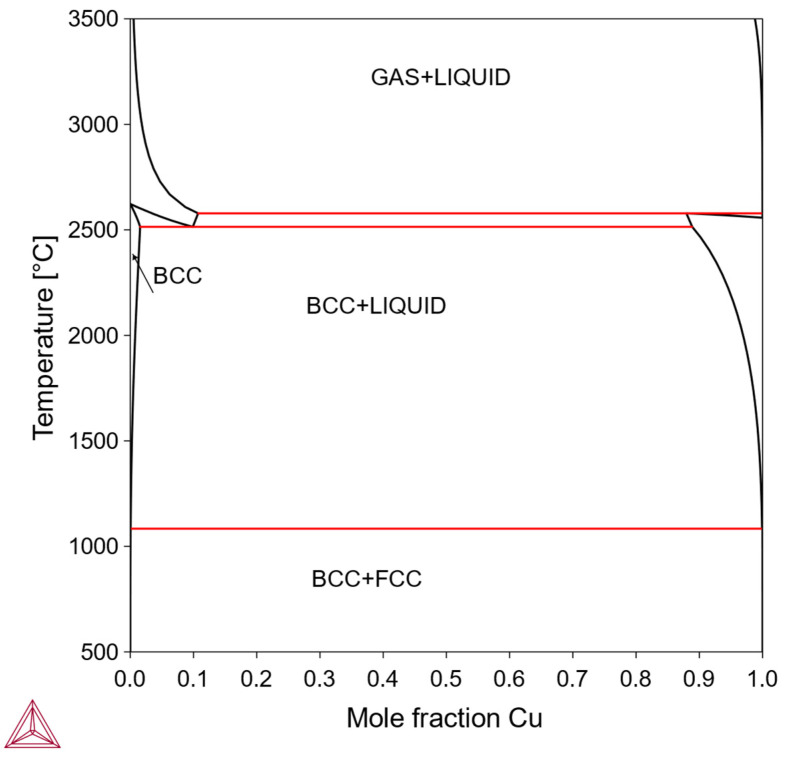
Mo-Cu phase diagram (Thermo-Calc).

Alloys of this type form the so-called “pseudo-alloys,” composed of an almost pure bcc Mo phase and an almost pure phase of X. Similar to Type IV, neither solid-solution strengthening nor precipitation strengthening is feasible. Some low-melting-point metal elements X may also cause processing difficulties. However, if some pure-X phase has good ductility and can form good interfacial bonding with bcc-Mo, the “pseudo-alloy” Mo-X may exhibit useful tailored mechanical properties between hard-and-brittle and soft-and-ductile. An example is the Mo-Cu system (Figure 5); since the fcc-Cu phase is relatively soft, has a suitable melting point, and is low cost, it could potentially improve toughness in the otherwise hard and brittle bcc-Mo matrix. However, Cu does not provide solid-solution strengthening or precipitation strengthening, due to its negligible solubility in bcc-Mo. Therefore, in this work we do not consider the Mo-Cu system and the other systems of Type V.

Based on the above classification, we infer that Types I, II, and III can be used for solid-solution-strengthened alloy design, and Type II could also be used for precipitation-strengthened alloy design. Types IV and V do not meet the conditions for either solid-solution (for two low solubility) or precipitation strengthening (for lacking a single-phase region for solutionizing). Therefore, elements of Types IV and V can be excluded from the design space of alloying compositions. The classification of alloying elements based on thermodynamic features is summarized in Table 2. Binary phase diagrams in Types I, II, III calculated using Thermo-Calc in this work are listed in the Supplementary Materials.

Dispersion strengthening, deformation strengthening, etc., are not further considered, because achieving high performance by these mechanisms relies mainly on processing technology, with composition and microstructure design playing a relatively minor role. Next, we are to focus on the candidate alloy systems for solution strengthening and precipitation strengthening, namely those belonging to Types I, II, and III. That leaves 28 Mo-X systems entering further investigation, which are X = Ti, V, W, Nb, Cr, Ta, Al, B, C, Co, Fe, Ga, Ge, Hf, Ir, Mn, N, Ni, Os, P, Pd, Pt, Re, Rh, Ru, Si, Zr, and Lu.

### 3.2. Potency of Precipitation Hardening

From the above classification, 21 elements (Al, B, C, Co, Fe, Ga, Ge, Hf, Ir, Mn, N, Ni, Os, P, Pd, Pt, Re, Rh, Ru, Si, and Zr) can serve as both solid-solution strengthening and precipitation strengthening alloying elements. In the same bcc Mo matrix, different precipitate phases can form depending on the alloy system. To quickly evaluate the strengthening capability of a precipitate phase, one can construct a metric for the potency of precipitation hardening based on fundamental theories of strengthening and phase transformations, in conjunction with thermodynamic calculations.

#### 3.2.1. Computational Model

**Figure 6:**
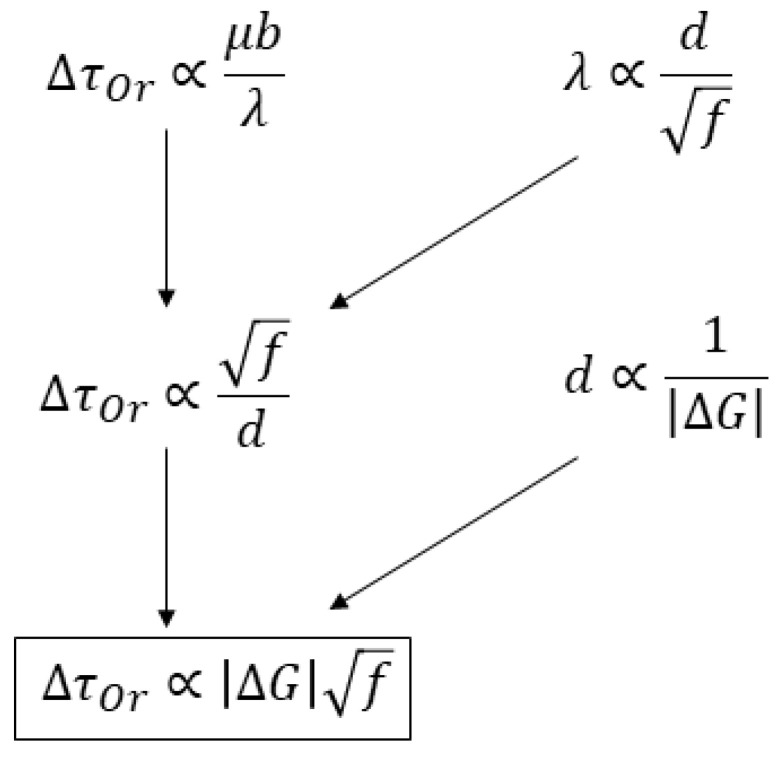
Metric for precipitation strengthening (redrawn after [28]).

Theories of precipitation strengthening are relatively mature. But many parameters within these theories can hardly be predicted from more fundamental mechanisms. In practice, the parameters are usually determined by fitting models to experimental data. In other words, given a specific matrix phase and a precipitate phase, it is currently difficult to predict the precipitation strengthening effect directly without experimental data. Nevertheless, based on the fundamental theory of second-phase precipitation and Orowan strengthening, it is possible to derive a metric that reflects the effect of precipitation strengthening, making it convenient to quickly evaluate various precipitate phases. The derivation process is illustrated in Figure 6. The reader can find a full description from the original reference [28].

The final equation suggests $\left|\Delta G\right|f^{1/2}$ is a reasonable metric for precipitation strengthening (MPS), where ΔG is the thermodynamic driving force of precipitation from supersaturated matrix phase, and f is the volume fraction of the precipitate after aging under a matrix-precipitate equilibrium. If the Mo-X binary phase diagram shows that more than one type of matrix-precipitate two-phase region is possible, then the precipitates are individually put into the calculation of matrix-precipitate stable and metastable conditions. Both ΔG and f can be obtained from thermodynamic calculations, making the evaluation of the MPS quite convenient in spite of a large number of alloy systems to be screened.

However, it should be noted that the Orowan equation is valid only for uniformly dispersed precipitates with small volume fractions. If the volume fraction is large, results from the Orowan equation are then only for a reference as the predicted strengthening may not be realized in practice. In such cases, a large fraction of a hard, brittle intermetallic, or ceramic precipitate would cause the material to fracture in a brittle manner, making it unsuitable for structural application.

#### 3.2.2. Evaluation Results for Binary Mo Alloys and Element Selection

**Figure 7:**
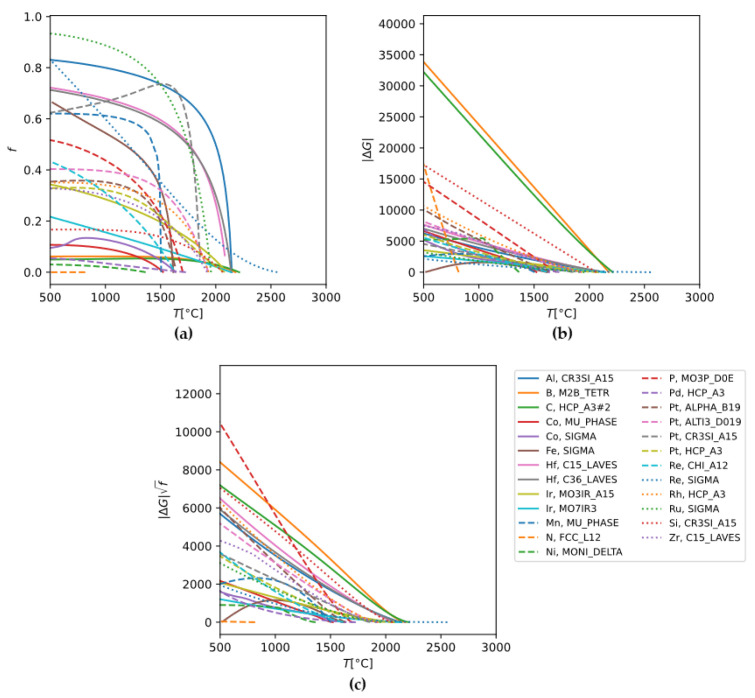
(**a**) Equilibrium volume fraction f, (**b**) precipitation driving force $\left|\Delta G\right|$, and (**c**) MPS $\left|\Delta G\right|\sqrt{f}$, evaluated for 18 Mo-X (X = Al, B, C, Co, Fe, Hf, Ir, Mn, N, Ni, P, Pd, Pt, Re, Rh, Ru, Si, and Zr) systems using Thermo-Calc.

At this point, MPS for 21 Mo-X binary systems are to be evaluated. A total of 18 of them can be performed directly because their Mo-X systems are assessed in available Thermo-Calc thermodynamic databases. To estimate the theoretically maximal strengthening effect, the calculations set the alloy composition to the maximum solubility of element X under equilibrium. The calculation results are shown in Figure 7. For practical applications, further considerations are needed, such as whether the volume fraction remains within the range of validity of the Orowan equation and whether a sufficient solution-treatment window exists. The actual precipitation-strengthening effect will be lower than the maximal values here.

Figure 7 shows the precipitate volume fraction, precipitation driving force, and MPS of the 18 Mo-X alloy systems. Some alloy systems have more than one precipitate phase, so for these systems, calculations are performed one by one with the bcc-Mo matrix. We observe that the phases with the highest $\left|\Delta G\right|$ are Mo_2_B, Mo_2_C, and Mo_3_Si. Mo_2_B and Mo_2_C have relatively low volume fractions, while Mo_3_Si has moderate phase fractions. In contrast, Mo_3_Al, Mo_3_P, Mo-Hf Laves phase and other intermetallic compounds have significantly lower driving force yet higher volume fractions. Introducing a large volume fraction of a hard intermetallic phase is detrimental to alloy performance. Also, the Orowan strengthening equation applies only to small precipitate volume fractions. Therefore, using the phases with low driving forces but high volume fractions for precipitate strengthening is not practical. Among the phases considered, Mo_2_B, Mo_2_C, and Mo_3_Si have moderately favorable volume fractions and high driving forces, making them representative precipitates for precipitation-strengthened binary Mo alloys.

## 4. Modeling Mechanical Properties of Solid-Solution Binary Molybdenum Alloys

Now we investigate 28 Mo-X alloy systems where solid-solution strengthening is possible. Models for mechanical properties (yield strength, ductility, ductile-brittle transition temperature DBTT, creep, and stress-strain curve) are briefly reviewed and then implemented to analyze Mo-X alloys theoretically or with respect to available experimental data.

### 4.1. Yield Strength

#### 4.1.1. Critical Resolved Shear Stress (CRSS) of Single Crystals

The theory of yield strength in bcc metals and alloys is relatively well-developed. Its basic framework is based on stress-assisted thermal activation theory and dislocation elasticity theory, analyzing the formation and migration kinetics of kinks on bcc screw dislocations [29,30,31]. Many theoretical models have evolved from this framework, differing primarily in the specific novel mechanisms proposed for the bcc screw dislocation (and even edge dislocations) and their corresponding barrier-driving-force relationships [32,33].

For pure Mo and binary Mo alloys, this project adopts the Trinkle-Woodward (TW) model [34] to calculate the single-crystal CRSS. The TW model is based on a thermally activated kink-pair formation mechanism on bcc screw dislocations, and it considers how the energy barrier for kink formation is affected by solute-dislocation interactions. The model parameters governing the strength are given as functions of the alloy composition.

**Figure 8:**
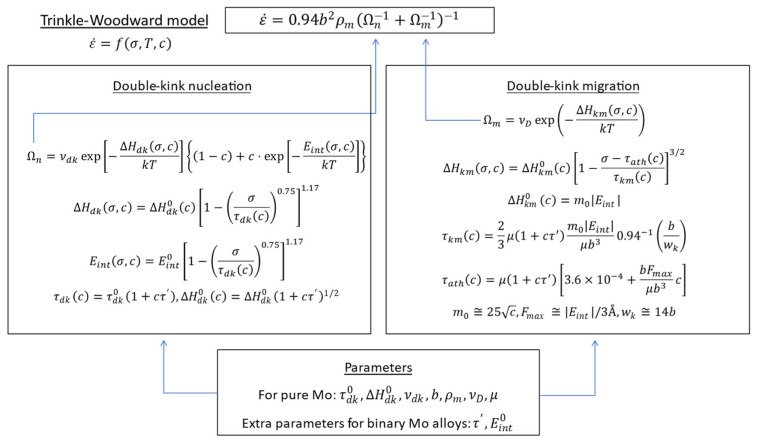
TW model for the CRSS of single-crystal binary Mo alloys (after [34]).

The TW model is elaborated in the original article [34]. Figure 8 demonstrates the calculation flow of the model.

This model eventually gives strain rate $\dot{\varepsilon }$ as a function of stress σ, temperature T, and concentration (atomic fraction) of alloying element, c. To determine the function for pure Mo, the parameters required are: b (Burgers vector), ρ (dislocation density), $\nu_{dk}$ (double-kink attempt frequency), $\Delta H_{dk}^{0}$, $\tau_{dk}$, $\nu_{D}$ (Debye frequency), and μ (shear modulus). These parameters are determined from fitting the model to experimental data of pure Mo single crystal. The parameters characterizing an alloying element are $\tau^{\prime }$, $E_{int}^{0}$. Therefore, to calculate yield strength of binary Mo-X alloys, it is necessary to obtain reasonable $\tau^{\prime }$ and $E_{int}^{0}$ values for the alloying elements under consideration.

To determine $\tau^{\prime }$ and $E_{int}^{0}$ for each element, Trinkle and Woodward in their original article [34] constructed a dislocation configuration in a pure bcc Mo matrix with the solute at various positions, and performed first-principles calculations. They determined necessary corrections to the first-principles results and provided values of $\tau^{\prime }$ and $E_{int}^{0}$ for six alloying elements (Re, Hf, Ta, Os, Ir, and Pt). Later, Yu et al. [35] provided the $E_{int}^{0}$ of Si in bcc-Mo also from first-principles calculations. However, the $\tau^{\prime }$ and $E_{int}^{0}$ for the other 21 alloying elements (Ti, V, W, Nb, Cr, Al, B, C, Co, Fe, Ga, Ge, Mn, N, Ni, P, Pd, Rh, Ru, Zr, and Lu) under consideration are still absent.

**Figure 9:**
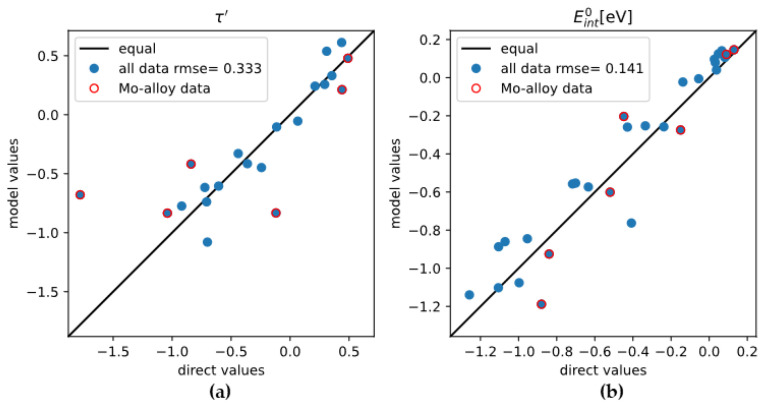
Performance of the polynomial model for (**a**) $\tau^{\prime }$ and (**b**) $E_{int}^{0}$, by comparing the model calculation results to the corresponding direct values from DFT calculations.

In this work, as an attempt to quickly estimate $\tau^{\prime }$ and $E_{int}^{0}$ for the other 21 alloying elements, we build a simple mathematical model based on the positions of alloying elements in the periodic table, with necessary adjustments based on experimental data [36]. Since first-principles results for alloying elements in bcc-Mo are scarce, we also adopt the first-principles results by Hu et al. [37], giving the $\tau^{\prime }$ and $E_{int}^{0}$ for 21 alloying elements in bcc-W using first-principles calculations. The model for $\tau^{\prime }$ or $E_{int}^{0}$ is written as a polynomial of Δp and Δg, which are the differences in Period number (Δp) and in Group number (Δg), respectively, between the alloying element and the matrix element (i.e., Mo or W). The model is as follows:

(1) $$y=a_{1}{\left(\Delta g\right)}^{3}+a_{2}{\left(\Delta g\right)}^{2}+a_{3}\Delta g+a_{4}\Delta p\Delta g+a_{5}{\left(\Delta p\right)}^{2}$$

where y stands for $\tau^{\prime }$ and $E_{int}^{0}$, $a_{1}\ldots a_{5}$ are fitting parameters. The polynomial satisfies the requirement that y=0 when Δp=Δg=0, i.e., when the “alloying element” is the matrix element itself. The data for W and the data for Mo are combined for model fitting. The optimized parameters are shown in Table 3, the performance of the model is demonstrated in Figure 9. The $\tau^{\prime }$ and $E_{int}^{0}$ values from the polynomial model and from first-principles calculations are listed in Table 4. For a few elements the values are adjusted manually to be more reasonable, according to experimental data on room-temperature hardness [36]. It should be explicitly pointed out that the polynomial model does not represent any physical significance. The model was fitted using mostly data about transition metals, so its predictability for main group elements, especially those occupying interstitial sites in bcc-Mo, can be low. The results are only used when first-principles calculation results are absent.

**Figure 10:**
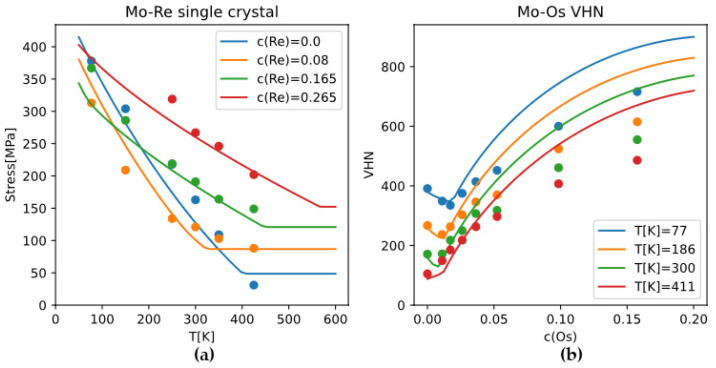
(**a**) CRSS of Mo-Re alloys and (**b**) VHN of Mo-Os alloys, experimental data (dots) vs. TW model calculations (lines). Alloys or conditions are distinguished by colors. Experimental data for Mo-Re and Mo-Os from References [38,39], respectively. Calculated VHN for Mo-Os is converted from CRSS using the relationship $VHN=44.8+1.203\times 10^{5}\left(\tau_{crss}/135GPa\right)$ given in Reference [34].

The calculated single-crystal CRSS of binary Mo alloys is a function of temperature, strain rate, and alloy composition. For Mo-Re, Mo-Os alloys of different compositions and temperatures, the TW model calculations agree with experimental data reasonably well (Figure 10). It can be seen from the plots that TW model gives an athermal “plateau” in strength-temperature relations, and the solute softening phenomenon in strength-concentration relations at relatively low temperature and low concentration (Figure 10). Both features are consistent with experimental data.

**Figure 11:**
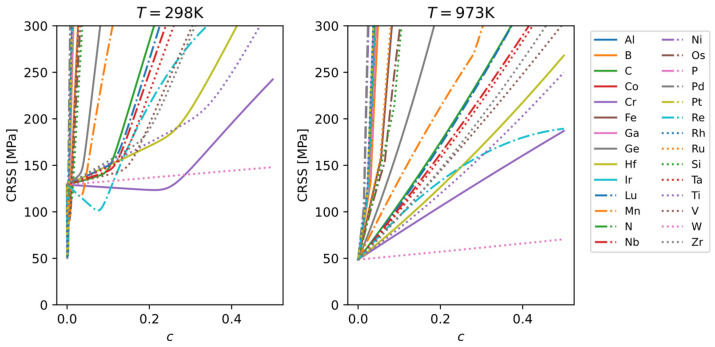
CRSS of Mo-X alloys at 298 K and 973 K, calculated using the TW model. Strain rate set to 10^−4^ s^−1^.

The single-crystal CRSS results for the binary Mo alloys from the TW model are shown in Figure 11. It can be seen that at room temperature, some alloying elements cause an initial solid-solution softening followed by strengthening, whereas at high temperature many elements show solid-solution strengthening. The efficiency of solid-solution strengthening (the slope of CRSS versus solute concentration) can vary significantly among different alloying elements.

#### 4.1.2. Yield Strength for Polycrystalline Alloys: Grain-Boundary Strengthening

Once the single-crystal CRSS is known, the yield strength of a polycrystalline aggregate can be obtained by considering the averaged effect of multiple activated slip systems. The result is a proportional relationship $\sigma_{y}=M\tau_{crss}$, where M is the Taylor factor. For an ideal polycrystal with randomly oriented, equiaxed grains, Kocks [40] gives M=3.06. Grain refinement further raises the polycrystalline yield strength according to the well-known Hall-Petch relationship:

(2) $$\sigma_{y}=M\tau_{crss}+\Delta \sigma_{HP}\;\Delta \sigma_{HP}=k_{HP}d^{-1/2}$$

Figueiredo and Langdon [41] considered grain-boundary sliding mechanisms at high temperature and extrapolated them to low-temperature regime. Based on this mechanism, they proposed the following formula:

(3) $$\Delta \sigma_{HP}=\sqrt{\frac{3GkT}{2db^{2}}\ln\left(\frac{\dot{\varepsilon }d^{3}}{10\delta D_{gb}}+1\right)}$$

where G is the shear modulus and $\delta D_{gb}$ is the product of the grain-boundary thickness and grain-boundary diffusion coefficient. One can see that this model does not strictly follow the original Hall-Petch $d^{-1/2}$ law, and it contains dependences on temperature T and strain rate $\dot{\varepsilon }$. Equation (3) has been applied to the literature data on fine-grained pure Mo and for pure Mo, $\delta D_{gb}\left(m^{3}/s\right)=5.5\times 10^{-14}\exp\left(-263000/RT\right)$ [41]. The Figueiredo-Langdon model for $\Delta \sigma_{HP}$ incorporates strain-rate and temperature dependences, which is already a step forward, compared to treating $k_{HP}$ as a fitting parameter without mechanistic understanding. The Figueiredo-Langdon model has not incorporated composition dependence yet, so for molybdenum alloys we use the values for pure Mo as an estimate.

**Figure 12:**
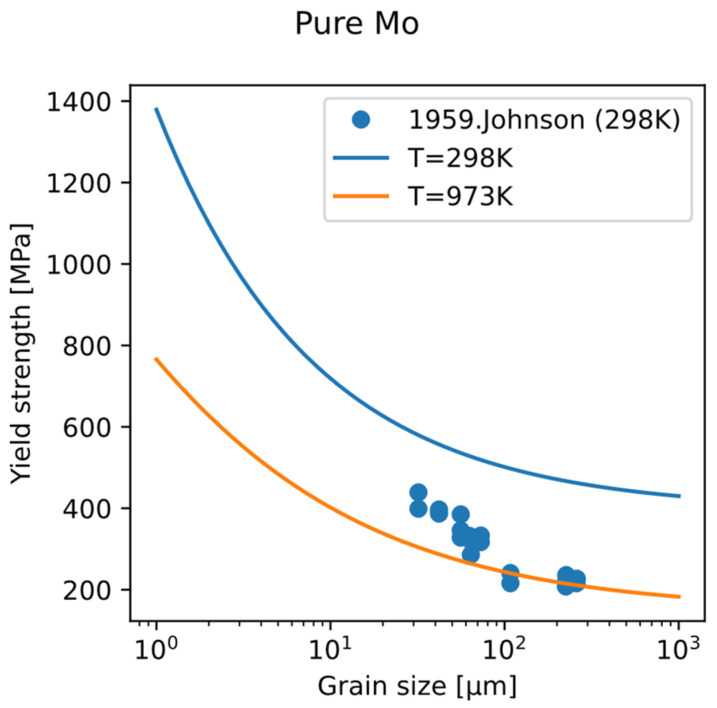
Relationship between the yield strength of polycrystalline pure Mo and its grain size, at 298 K and 973 K. Strain rate set to 10^−4^ s^−1^. Experimental data at room temperature (dots) are from Reference [42].

Using this fine-grain strengthening model, the variation in yield strength of polycrystalline pure Mo with grain size at room temperature and 700 °C was calculated (Figure 12). It can be seen that as the grain size decreases, the yield strength increases. The effect of grain refinement weakens with the increase in temperature. The experimental data of the relationship between yield strength and grain size show the same trend. Therefore, grain refinement provides an independent direction from solid-solution strengthening and precipitation strengthening, which is not dependent on composition design and is helpful in reaching design objective for strength.

#### 4.1.3. Effect of Alloying Elements on CRSS or Yield Strength

Experimental data on alloy hardness or strength for different alloying elements allow us to rank and classify the relative efficiency of solid-solution strengthening. According to Figure 11, the strengthening efficiency of various elements at 25 °C varies over a wide range and can be roughly categorized as follows:

- Very high (+++): Pd, Ni, Rh, Pt, Ga, Co, N, Ir, Al, P, and B.
- Medium high (++): Ru, Fe, Os, Si, Ge, Mn, C, Lu, Nb, Ta, V, Zr, Re, Hf, and Ti.
- Low (+): Cr and W.

From this classification, we observe the following general trends:

- Very high strengthening effect: mainly noble metals and interstitial elements.
- Medium-high strengthening effect: other transition metals (not in the same group as Mo), main-group metals, etc.
- Low strengthening effect: elements in the same group as Mo.

### 4.2. Ductility

**Figure 13:**
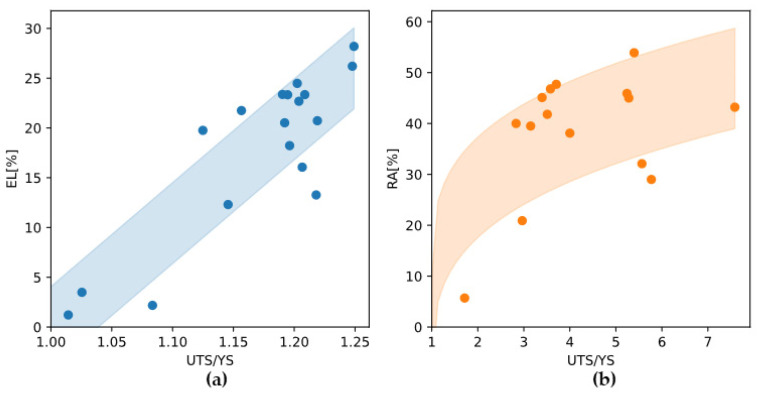
Ductility in terms of total elongation (EL, %) or reduction in area (RA, %) correlated to the ratio of UTS to YS. Experimental data are from (**a**) Mo-X (X = W, Nb, Ta, Ti) alloys (room temperature) [43] and (**b**) Mo-X (X = Cr, Re, Ta, Ti, W) alloys (500 °C) [44].

From a stress-strain curve one can see that after yielding, the alloy undergoes plastic deformation and requires work hardening before reaching the ultimate tensile strength (UTS). If an alloy has very poor ductility or fractures in a brittle manner, the work hardening process is not fully realized, which is detrimental to the increase in strength from yield to UTS. Plotting experimental data shows that both total elongation and reduction in area are positively correlated with the ratio of UTS to yield strength (Figure 13). Therefore, in addition to yield strength, improving the ductility of the alloy is an important way to ensure excellent tensile strength.

**Figure 14:**
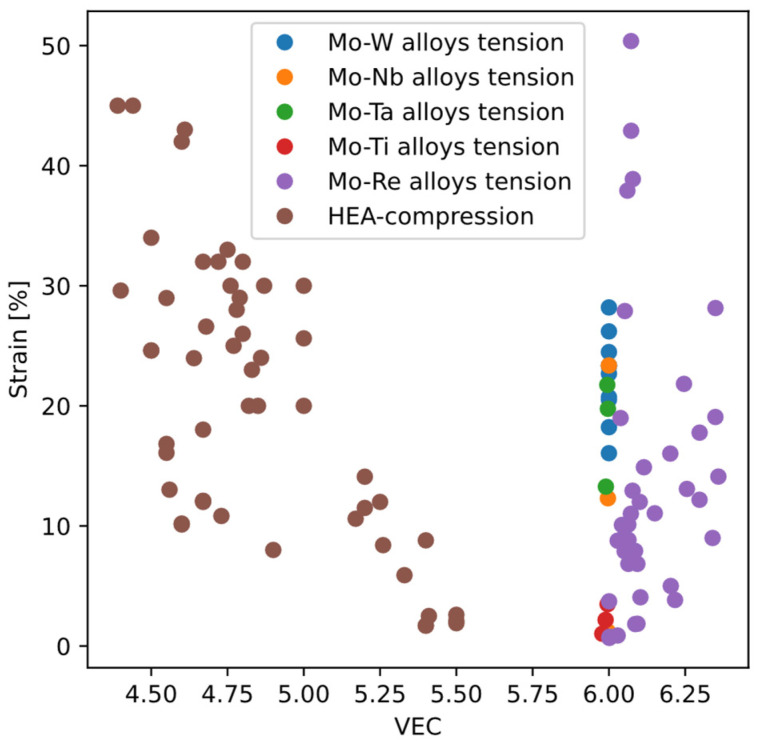
Ductility of Mo alloys vs. VEC, as compared to bcc high-entropy alloys (HEA) vs. VEC. The Mo-alloy ductility data are taken from [43,46] including Mo-X (X = W, Nb, Ta, Ti, Re) alloys. The HEA ductility data are taken from compiled data in [45], measured by fracture strain from compression test.

In contrast to yield strength which can be modeled by analyzing one dislocation, ductility involves complex mechanisms of dislocation interactions, even including damage, making it very difficult to predict mechanistically. Therefore, researchers have proposed a number of metrics that can be correlated to ductility. For example, Singh et al. [45] summarized previous empirical metrics for ductility, including (1) valence electron concentration (VEC), (2) Cauchy pressure $C_{12}-C_{44}$, (3) Pugh ratio B/G, (4) “D parameter” $\gamma_{s}/\gamma_{usf}$. Singh et al. also propose a new metric “LLD” based on atomic displacements between the ideal bcc structure and the relaxed, equilibrium positions of atoms. Except the “D parameter”, these metrics are all about properties of perfect bcc structure, i.e., without any dislocations or other crystal defects, presumably because they are convenient to calculate or measure. However, among the metrics based on perfect-crystal properties, different levels of predictability can be observed. Taking VEC as an example (Figure 14), for high-entropy alloys (HEA), ductility tends to decrease with increasing VEC; but for molybdenum alloys the opposite holds.

Our motivation is to find a metric for ductility based on perfect crystal structure (free from dislocations and other crystal defects) for convenience. Building on previous work that considered lattice distortion [45] and defect energetics in solid solutions, and aiming to further explore the construction of physically meaningful plasticity indices without excessive computational cost, this project proposes a new plasticity indicator that takes into account both the structure and energetics of bcc solid solutions.

We calculated the equilibrium lattice constants of these bcc supercells, i.e., the atomic relaxation displacements and the associated energy change. We extract two variables from supercell relaxation:

- Average atomic displacement magnitude: the magnitude of atomic displacements from the ideal bcc lattice positions to the equilibrium positions, then averaged over all atoms. This has been used by Borges et al. [47] also for the purpose of analyzing ductility.
- “Average bond stiffness”: the relaxation energy divided by the “average atomic displacement magnitude” squared.

Among the alloys, we infer that smaller average displacement (i.e., the preservation of bcc lattice symmetry) is favorable to dislocation motion and therefore to ductility; similarly, weaker bonds between atom also favor ductility, meaning less force and energy are required to move atoms locally.

**Figure 15:**
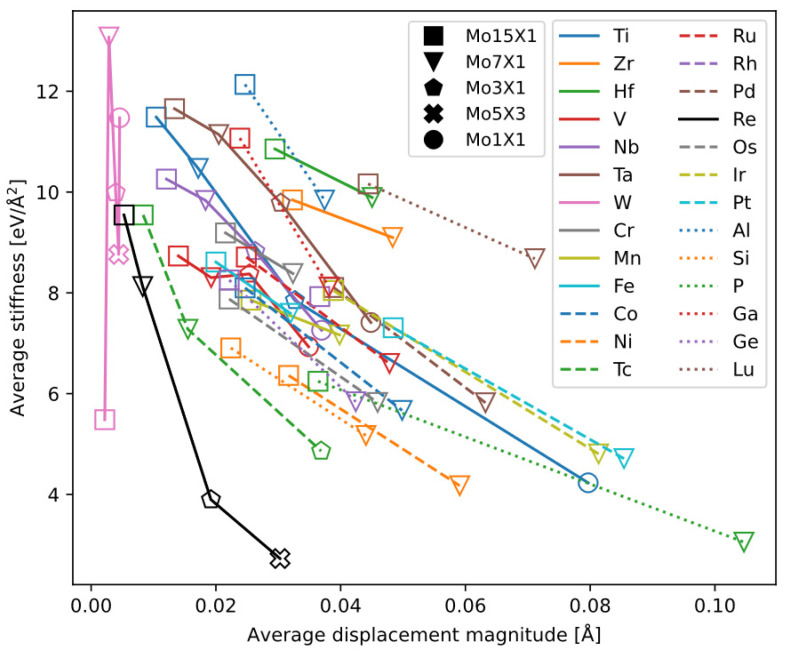
Average displacement magnitude (x-axis) vs. “average bond stiffness” (y-axis) for bcc Mo-X alloys. Alloying elements are specified by color and line style; concentration is specified by shape of the markers.

Plotting the two indicators calculated for each alloy against each other allows us to analyze the characteristics of the alloying elements (Figure 15). The general trend is that as the solute concentration increases, the average atomic displacement increases while the “average bond stiffness” decreases. Curves of most elements cluster together in a band, whereas a few elements exhibit distinct behaviors. By classifying the elements, we summarize the following trends:

- W causes the smallest average atomic displacement among the elements studied. Although the Mo-W data show some scatter, generally they lie near the starting points of the other alloy series at low concentration.
- Re causes the largest reduction in “average bond stiffness” among the elements studied, and it also causes a relatively small average atomic displacement at the same concentration (second only to W). Compared to many other elements, Re exhibits a uniquely favorable trend for ductility. Thus, from the perspective of average atomic displacement and “average bond stiffness,” our results provide confirmation of the unique “Re effect.”
- Tc is in the same group as Re, and its effect is close to that of Re, in maintaining small atomic displacement and reducing average bond stiffness. However, calculating Mo-Tc alloys is only for theoretical interests. Tc has been excluded in this work as an alloying element of Mo, due to its scarcity and radioactivity.
- The four elements Ti, V, Nb, and Ta, which are all infinitely soluble in bcc Mo, excepting W and Re, also cause relatively small displacements at low concentrations (except for Mo_1_Ti_1_). Among these four, their “average bond stiffnesses” follow the order V < Nb < Ti < Ta.
- Zr, Hf, and Lu lie in the upper-right region of the plot, suggesting they are likely detrimental to ductility. Curves of the remaining transition-metal elements together with some main group elements are clustered in a band, making it difficult to identify any elements other than Re that might benefit ductility.

From the calculated metrics, we did not find another element with Re’s unique behavior in bcc-Mo. For the other alloying elements, systematic experimental data are lacking, making it difficult to validate the relationship between the metrics and experimental ductility data. A more detailed validation of the metrics using other bcc alloys is to be published [48].

### 4.3. Ductile-Brittle Transition

The phenomenon where metals and alloys exhibit significant reduction in plasticity or toughness with decreasing temperature, manifesting as brittleness, is generally termed as ductile-brittle transition (DBT). This is a common feature of BCC metals and alloys including molybdenum and its alloys. When the ductile-brittle transition temperature (DBTT) is higher or close to room temperature, it adversely affects the workability of alloys at room temperature. Therefore, lowering DBTT becomes necessary to ensure adequate room-temperature processability.

Strictly speaking, ductility and toughness represent distinct mechanical properties: ductility quantifies the magnitude of achievable deformation, while (fracture) toughness measures the resistance to crack initiation and propagation, or the energy dissipation during fracture. Therefore, DBTT is often characterized through temperature-dependent variations in various indicators, including (1) total elongation and reduction in area under uniaxial tension; (2) minimum bending radius or maximum bending angle for plates/rods; (3) quasi-static fracture toughness parameters (K or J); and (4) Charpy impact toughness. Among these, uniaxial tensile tests and fracture toughness measurements offer relatively simple mechanical states and better standardization, though reported data remain limited. Bending and impact tests find wider engineering applications due to their simplicity, but suffer from analytical complexity and potential methodology-dependent data variations.

Mathematical models for describing the transition are commonly employed in engineering practice. For sparse plasticity/toughness-temperature data, empirical formula fitting may be applied [49]. When sufficient data exist to reveal probabilistic distributions, the master curve method enables statistical analysis [50]. The fitted parameters represent material-specific transition characteristics, enabling direct comparisons between materials under identical testing and analysis protocols. These data processing approaches disregard microstructural variables or other mechanical properties, thus requiring minimal materials science knowledge.

Theoretical understanding and mathematical modeling of the transition based on materials science principles remains feasible. According to Armstrong’s analysis [51], the ductile-brittle transition occurs when the temperature-dependent yield strength $\sigma_{y}$ exceeds a critical “brittle fracture strength” $\sigma_{c}$. As $\sigma_{y}$ can be measured above DBTT and $\sigma_{c}$ below DBTT, and considering existing theoretical models for $\sigma_{y}$ (Section 4.1), Armstrong’s framework provides a convenient physical interpretation of the transition. Effects of testing conditions and microstructure on DBTT can be analyzed through their respective influences on $\sigma_{y}$ and $\sigma_{c}$. Therefore, subsequent analyses preferentially utilize uniaxial tensile data to avoid mechanical complexities inherent to bending and impact testing methodologies.

#### 4.3.1. Grain-Size Dependence of DBTT

Based on the summary of Armstrong, the $\sigma_{y}$ and $\sigma_{c}$ can both follow the Hall-Petch relationship:

(4) $$\sigma_{i}=\sigma_{i0}+k_{i}d^{-1/2},\;i=y,c$$

**Figure 16:**
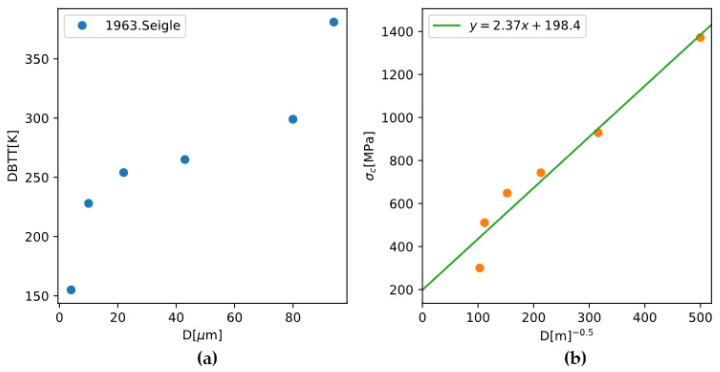
(**a**) Experimentally measured DBTT for pure Mo [53]; (**b**) brittle fracture stress back-calculated from DBTT and yield strength modeled in Section 4.1 (dots), and a linear regression (line) to the points.

The experimental observation that smaller grain sizes lead to lower DBTT can be rationalized by letting $k_{c}>k_{y}$. Given the experimental data for the grain-size dependence of DBTT of polycrystalline molybdenum and with the $\sigma_{y}$ calculated using the TW model, $\sigma_{c}$ and its dependence on grain size can be back-calculated (Figure 16). The results give $k_{c}=2.37\;MPa\cdot m^{1/2}$ which is comparable to the previous report $k_{c}=2\;MPa\cdot m^{1/2}$ [52].

#### 4.3.2. Strain-Rate Dependence of DBTT

**Figure 17:**
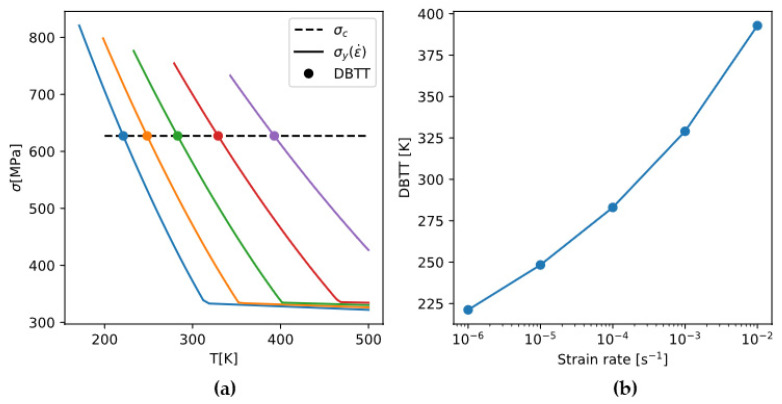
(**a**) Yield strength of pure Mo at different strain rates (solid lines in colors, from left to right: 10^−6^, 10^−5^, 10^−4^, 10^−3^, 10^−2^ s^−1^ respectively) intersecting a constant brittle fracture stress $\sigma_{c}$ (dashed lines), resulting in a strain-rate-dependent DBTT (**b**).

It is experimentally shown that $\sigma_{c}$ does not vary significantly with temperature or strain rate [54]. Therefore, the strain-rate dependence of DBTT is brought about solely by that of $\sigma_{y}$. Figure 17 demonstrates that the rate-sensitive yield strength $\sigma_{y}$ intersects the rate-insensitive brittle fracture stress $\sigma_{c}$ at different temperatures (DBTT), giving a strain-rate dependence of DBTT.

#### 4.3.3. Composition Dependence of DBTT

The analysis of DBTT of molybdenum alloys is, in principle, not different from that of DBTT of pure molybdenum. The effect of composition on DBTT can still be analyzed through the effects on yield strength and brittle fracture strength. But in practice, there is much less data about DBTT of molybdenum alloys than data about DBTT of pure molybdenum. In some reports, DBTT of alloys are only correlated to the overall composition, without considering possible changes in microstructure.

**Figure 18:**
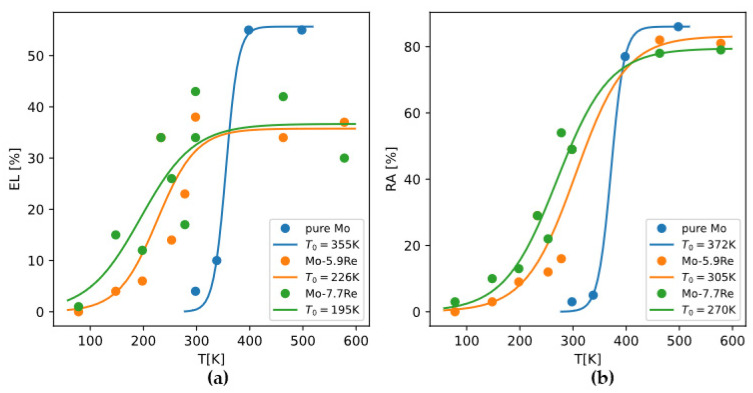
Curve fitting ductility-temperature relationship to extract DBTT using (**a**) total elongation or (**b**) reduction in area. Experimental data from Reference [55].

**Figure 19:**
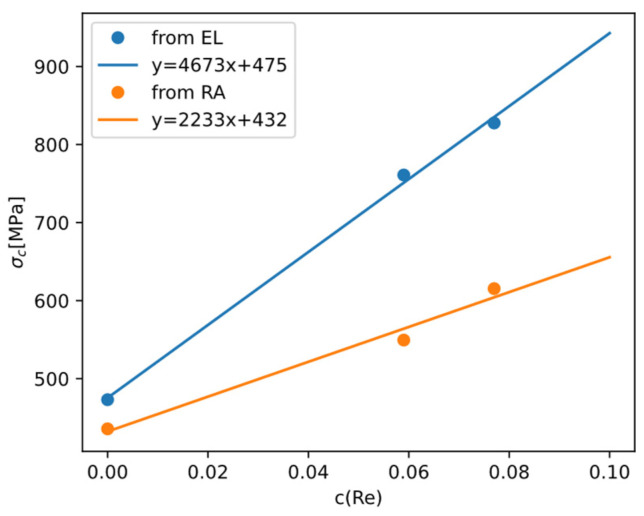
Brittle fracture stress as calculated using DBTT from total elongation (Figure 18a) or DBTT from reduction in area (Figure 18b).

Because there are relatively more data about yield strength ($\sigma_{y}$) and DBTT of Mo-Re alloys, we can invoke Armstrong’s theory and derive how $\sigma_{c}$ changes with Re content. Reference [55] reported for alloys Mo-xRe (x = 0, 5.9, 7.7at%) alloys how total elongation (EL) and reduction in area (RA) change with temperature. Using a master curve $y=y_{0}\left\{1+\tanh\left[\left(T-T_{0}\right)/\Delta T\right]\right\}/2$ to fit the data, we can take the optimized $T_{0}$ as the DBTT (Figure 18). Then because $\sigma_{y}=\sigma_{c}$ holds at DBTT, and $\sigma_{y}$ of polycrystalline Mo alloys can be readily calculated based on the models in Section 4.1, we can back-calculate $\sigma_{c}$ and examine how it depends on Re content (Figure 19).

From the results we can see Re is effective in raising $\sigma_{c}$, which is the main reason why Re is effective in lowering DBTT while raising yield strength $\sigma_{y}$.

### 4.4. Creep

In solid solutions, the rate-controlling mechanism of dislocation creep is vacancy diffusion; that is, the dislocation climb rate is controlled by the rate at which vacancies arrive at or depart from the dislocation core. According to this mechanism, the steady-state creep rate of a single-phase alloy can be expressed [56] as

(5) $${\dot{\varepsilon }}_{ss}=A{\left(\frac{\sigma }{G}\right)}^{n}Gb\frac{D_{Va}}{k_{B}T}$$

where $D_{Va}$ is the vacancy diffusion coefficient, and the other terms are: temperature T, applied stress σ (usually assumed to be uniaxial tension), shear modulus G, stress exponent n (for dislocation glide or climb mechanisms, n ≈ 3~5), Burgers vector b, Boltzmann’s constant $k_{B}$, and a material constant A. For pure metals, $D_{Va}$ is equal to the self-diffusion coefficient. For a solid solution, $D_{Va}$ can be obtained by a weighted average of the self-diffusion or impurity diffusion coefficients of all substitutional elements [57].

(6) $$D_{Va}={\left(\sum_{i\in S}x_{i}D_{i}^{-1}\right)}^{-1}$$

Consequently, one way to improve the creep resistance of the solid solution is to add alloying elements with low diffusion coefficients.

**Figure 20:**
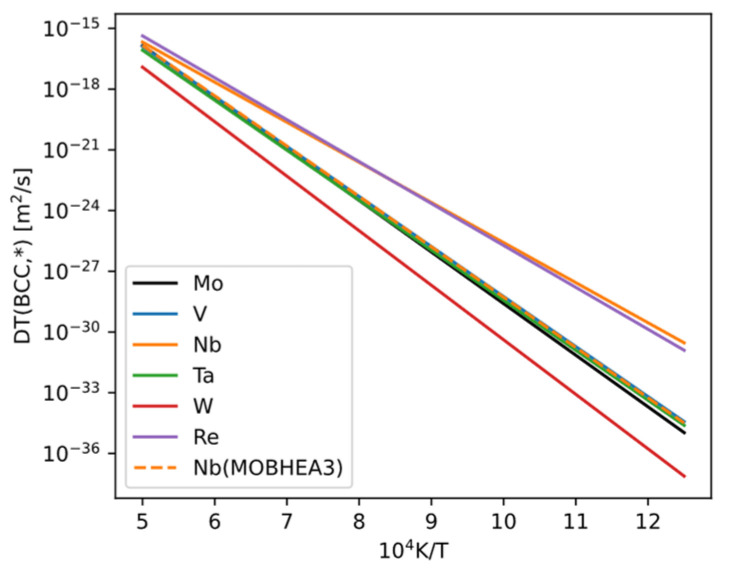
Tracer diffusivities of some slow-diffusing alloying elements (colored lines) in bcc-Mo, compared with the self-diffusivity of pure Mo (black line). Results for all elements are from MOBNI5 (by default), and Nb from MOBHEA3 is also shown.

The self-diffusion coefficient of bcc Mo and the impurity diffusion coefficients of solutes in bcc Mo depend not only on temperature but also on the composition of the bcc phase. Generally, many elements have measured impurity diffusion coefficients, whereas fewer have measured chemical diffusion coefficients (which depend on composition). Both self-diffusion and impurity diffusion coefficients can be obtained from mobility databases (e.g., MOBNI5, MOBHEA3 in Thermo-Calc), but the values may be uncertain (due to experimental uncertainties or lack of data, sometimes estimated by database authors). We compiled the self-diffusion and impurity diffusion values in pure bcc Mo (Figure 20) and found the following: among the elements included in the database that we can compute, only W has a diffusion coefficient lower than that of Mo. The values for V, Nb, Ta, and Re are close to or slightly higher than Mo. Apart from a discrepancy for Nb, the values for these elements are the same in both MOBNI5 and MOBHEA3 databases. We also checked all other elements in the database and found that their impurity diffusion coefficients are all higher than the self-diffusion coefficient of Mo. We infer that these elements would not be beneficial for improving creep resistance.

### 4.5. Stress-Strain Curves

The methods of modeling stress-strain curves range from completely empirical models [58] to detailed mechanistic models with internal state variables [59]. An intermediate choice of stress-strain model is the Zerilli-Armstrong (ZA) model [60,61,62] which incorporates temperature- and strain-rate-dependences of stress-strain curves. It has some physical significance and a moderate number of fitting parameters.

A relatively well-developed version is (for bcc metals and alloys)

(7) $$\sigma =\sigma_{G}+B\exp\left(-\beta T\right)+B_{0}\sqrt{\varepsilon_{r}\left[1-\exp\left(-\varepsilon /\varepsilon_{r}\right)\right]}\;\beta =\beta_{0}-\beta_{1}\ln\dot{\varepsilon }$$

$\sigma_{G}$ is a “baseline” strength at T=0 and ε=0, which can also decompose into contributions from solid-solution strengthening, grain-boundary strengthening, etc. For a set of stress-strain curves of the same material but tested under different temperatures and strain rates, ideally one ZA model can be fit to all the stress-strain curves, using six parameters: $\sigma_{G},\;B,\;\beta_{0},\;\beta_{1},\;B_{0},\;\varepsilon_{r}$. If the ZA model parameters are known, tensile strength and uniform elongation can be found at the condition dσ/dε=σ.

Stress-strain curves of pure Mo and Mo-Re alloys are relatively systematically reported in the literature, compared to the scarcity of stress-strain curves of other binary alloys. In this work, we use ZA model to fit experimental stress-strain curves of pure Mo and Mo-Re alloys, and discuss the effect of Re content on ZA model parameters.

**Figure 21:**
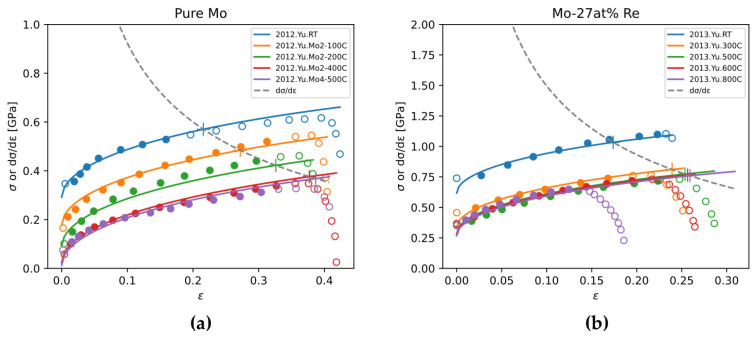
Fitting ZA model to the stress-strain curves of (**a**) pure Mo and (**b**) Mo-27 at% Re. Experimental data from [63,64]. Solid dots are utilized for fitting, while open circles are not. Testing conditions are distinguished by colors.

**Figure 22:**
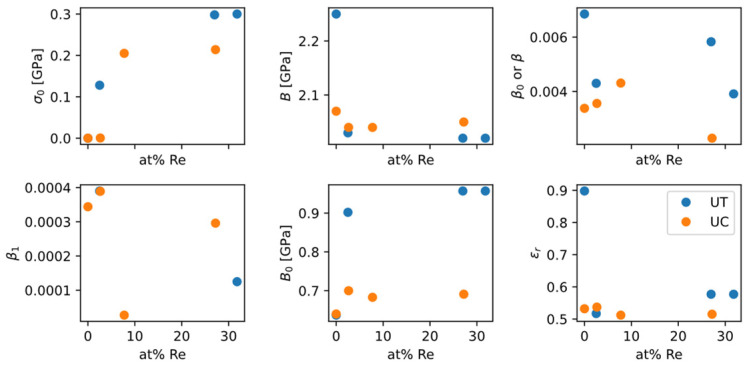
ZA model parameters changing with Re content in Mo-Re alloys. Experimental stress-strain data from References [63,64,65,66,67]. Blue dots for uniaxial tension (UT) and orange for uniaxial compression (UC).

The sources of experimental data for fitting are from References [63,64,65,66,67]. Typical fitting results are shown in Figure 21. Because the raw data of stress-strain curves were not published, data points were read from the plots of original publications with reasonable intervals. The obtained ZA model parameters are plotted in Figure 22.

In spite of the significant scattering, the data from Figure 22 show some qualitative trends:

- $\sigma_{0}$ as a “baseline” strength increases as Re content increases, which is consistent with the solid-solution strengthening effect of Re;
- $B_{0}$ as a measure of strain-hardening magnitude slightly increases as Re content increases, which is favorable for ductility, and is consistent with the “rhenium effect” in increasing ductility;
- Tension/compression difference: while $\sigma_{0}$ does not exhibit much difference between tension and compression, $B_{0}$ from tension and $B_{0}$ from compression have a large gap in between. It suggests that when studying how ZA model parameters change with alloy composition, experimental data from different types of tests should not be mixed together and used for fitting.

The ZA model fitting plays an explanatory role. It is used after (not before) an experimental stress-strain curve has been obtained, as a method of digitizing and storing the stress-strain curve to support various types of simulations that require materials properties.

### 4.6. Summary of Binary Mo-X Systems

Based on the modeling work in this section, we can summarize the effects of 28 alloying elements for solid-solution strengthening on the mechanical properties analyzed in this section:

- Yield strength: All elements can provide solid-solution strengthening with various efficiency. At room temperature there are some elements exhibiting the “solute softening” phenomenon, but it can be overcome by increasing alloying element content.
- Ductility (tensile strength to yield strength ratio): Based on the ductility metric calculations in this work, only Re exhibits outstanding features as a commonly used alloying element that is favorable to ductility. Ductility metrics of a large number of alloying elements are clustered, not showing a similar behavior to Re.
- Creep: One way to increase creep resistance for solid-solution alloy is to lower the vacancy diffusivity in the alloy by introducing slow-diffusing alloying element. From mobility databases, the only diffusant known to be slower than Mo is W. But the tracer diffusivities of V, Nb, Ta, and Re are close or slightly higher than the self-diffusivity of Mo.
- DBTT and stress-strain curve modeling: The modeling approaches adopted in this work are able to capture their dependences on some conditions or materials parameters, such as grain size (for DBTT), strain rate (for DBTT and stress-strain curve), and temperature (stress-strain curve). However, the modeling approach are explanatory, rather than predictive, in the chemical composition space. Therefore, this part of the work does not play a central role on optimizing the choice of alloying elements.

## 5. Discussion

### 5.1. Priorities of Mechanical Properties of Mo Alloys for High-Temperature Applications

Comparing the mainstream Ni-base superalloys, Mo alloys are advantageous in high-temperature strength and creep resistance, due to its bcc crystal structure and a higher melting temperature. But Mo alloys are disadvantageous in low-temperature ductility, which can cause problems in Mo alloy fabrication and processing. If the purpose is to search for an alloy that preforms like Ni-base superalloys, the guidelines include (ranked in priority):

- Increase alloy ductility;
- Solid-solution strengthening to some extent, exploring grain-boundary strengthening or work hardening when necessary;
- Avoid introducing impurities which embrittles the alloy to maintain a reasonable DBTT;
- Slight deterioration of creep resistance is tolerable.

Based on the list above and the work presented in Section 4, the most promising alloying strategy is adding Re to a sufficient amount, because Re plays a unique role in enhancing ductility in Mo alloys. According to the ductility metric calculation results, no other element among the 28 elements for solid-solution strengthening exhibit a similar trend to Re.

For strengthening, the choices are plenty because all of the 28 elements increase yield strength. Also there are grain-boundary strengthening and work-hardening, etc., as other ways to increase strength. So there is not a strong preference in selecting alloying elements for strengthening from a technical perspective.

About creep, diffusivity data shows that W diffuses slower than Mo, and Re is among the slowest diffusers. Based on the results, W is more advantageous than Re as an alloying element to enhance creep resistance. However, since creep resistance is not a top priority, and W does not increase ductility of Mo alloys, Re remains the most advantageous as an alloying element in binary Mo alloys.

### 5.2. Uncertainties and Gap Analysis

In general, uncertainties in modeling and calculations may affect optimization of alloy composition. Here we briefly discuss different sources of uncertainties involved in this work, and how they affect the conclusions.

About binary phase diagrams and thermodynamics, there are many Mo-X binary systems not covered by CALPHAD databases, for which handbooks of phase diagrams are used. Both CALPHAD assessments and handbook phase diagrams have uncertainties. When necessary, sources of experimental data used to construct the CALPHAD database or the phase diagram should be traced and analyzed. In this work, many Mo-X binary systems not yet assessed in the CALPHAD way are in Type IV and Type V, i.e., systems with negligible solubility of X in bcc-Mo. But both types have been excluded from further considerations; therefore, uncertainties in these types of phase diagrams do not affect our conclusion. Precipitation-strengthening alloys are the most sensitive to uncertainties in thermodynamics, because they require a solutionizing in single-phase region and an aging treatment in two-phase region. The solubility curve is key information for determining the heat treatment scheme, therefore the uncertainty in it can significantly affect the success rate of heat treatment. However, solid-solution strengthened alloys usually do not require much heat treatment, making them insensitive to uncertainties in phase boundaries. Therefore, the impact of uncertainties in phase diagrams and CALPHAD databases depends on the type of alloy system under consideration.

About mechanical properties, the amount of available experimental data for the 28 Mo-X systems that can be used for solid-solution strengthening (including the 21 Mo-X systems that can also be used for precipitation strengthening) varies greatly among alloy systems; Mo-Re alloys are relatively thoroughly investigated, whereas there are much fewer data available about other alloys and they are often limited to dilute concentrations. This situation certainly affects the judging of the effect of alloying elements on strength, ductility, DBTT, etc. In this case, some mechanical properties may find inputs from calculations on a more fundamental level. A typical case is the use of the TW model and how first-principles calculations can determine model parameters for a new alloying element. Currently there are only seven elements that have available TW model parameters. In this work, TW model parameters for the other alloying elements are estimated using a polynomial model with the help from results of W-base alloys. The estimated TW model parameters and their predictions of yield strength must be validated by more first-principles calculations or experimental measurements. The model for DBTT and the ZA model for stress-strain curves are descriptive rather than predictive. In this case, the quality of fitting is determined by the quality of available experimental data, and their uncertainties are closely related.

## 6. Conclusions

Based on thermodynamic calculations and the literature survey for phase diagrams, binary Mo-X systems are categorized into five types based on solubility of X in the bcc-Mo matrix and existence of intermediate phases. Alloy systems Mo-X where X has negligible solubility in bcc-Mo, or when X is a radioactive element, are excluded from further consideration. A total of 28 alloy systems remain and are further investigated about mechanical properties.

Among the 28 alloy systems, 21 have stable intermediate phases, making them possible to be utilized for precipitation strengthening. The objective is to produce a large number density of fine particles of a precipitate phase. A computational model based on thermodynamics, kinetics, and strength theory is used to quickly evaluate the potency of different precipitate phases in alloy systems. The results suggest that Mo-B, Mo-C, Mo-Si alloy systems are the most promising candidates in delivering high precipitation strengthening.

The 28 alloy systems with continuous bcc phase region or limited solubility are further evaluated as solid-solution strengthening alloys in terms of yield strength, ductility, DBTT, creep resistance, and stress-strain curves. Mechanistic models supported by experimental data from the literature or by first-principles calculation are adopted to evaluate different alloy systems. The results show that a large number of alloying elements can provide solid-solution strengthening. Re is unique in its ability to improve the ductility of Mo alloys, supported by first-principles calculations of the atomic structure and energetics of Mo-Re solid solutions. W is unique as a slow-diffusing element which can improve creep resistance. DBTT and stress-strain curves are modeled and calibrated using available experimental data. Currently, models for DBTT and stress-strain curves are explanatory but not predictive for the purpose of composition optimization.

Mo alloys for high-temperature applications, in comparison to Ni-base superalloys, are disadvantageous in ductility, but advantageous in strength and creep resistance. Since Re is the only element known to improve the ductility of Mo alloys, Mo-Re is the most promising alloy system for ductile, high-temperature alloys.

This work is a case study demonstrating how different modeling and computational methods can be integrated to investigate phase relations and mechanical properties of a large number of molybdenum alloys. The workflow in this work can also be applied to other bcc refractory alloys. Future directions may include selecting/designing multicomponent, multiphase refractory alloys, with combined approaches for strengthening and ductilizing.

## Figures and Tables

**Table 1:** Sources of Mo-X binary phase diagrams.

Source	Elements X	Quantity
TCNI10	Al, B, C, Ca, Co, Cr, Cu, Fe, Hf, Mg, Mn, N, Nb, Ni, O, Pd, Pt, Re, Ru, S, Si, Ta, Ti, V, W, Y, Zr	27
TCTI5	H	1
TCHEA6	Ir, Rh, Zn	3
TCFE10	Ce, P	2
Assessments from the literature	La [22], Sn [23,24], Yb [25]	3
Handbook [26]	Ag, As, Au, Ba, Be, Bi, Br, Cd, Cl, Cs, Dy, Er, Eu, F, Ga, Gd, Ge, Hg, Ho, I, In, K, Li, Lu, Na, Nd, Os, Pb, Pr, Rb, Sb, Sc, Se, Sm, Sr, Tb, Te, Tl, Tm	39

**Table 2:** Types of Mo-X binary alloy systems based on thermodynamic features.

Type	Elements X	Quantity	Comments
I. Continuous bcc-Mo-X solution	Ti, V, W, Nb, Cr, Ta	6	Solution-strengthening
II. Finite solubility of X in bcc-Mo, with intermediate phase(s)	Al, B, C, Co, Fe, Ga, Ge, Hf, Ir, Mn, N, Ni, Os, P, Pd, Pt, Re, Rh, Ru, Si, Zr,	21	Solution-strengthening and precipitation are both possible
III. Finite solubility of X in bcc-Mo, without intermediate phases	Lu	1	Solution-strengthening
IV. Negligible solubility of X in bcc-Mo, with intermediate phase(s)	Be, Zn, Sn, As, Sb, O, S, Se, Te, F, Cl, Br, I	13	Not practical for solution- or precipitation-strengthening, embrittling, not considered further
V. Negligible solubility of X in bcc-Mo, without intermediate phases	Bi, La, Ag, Ca, Y, Au, Cd, Cs, Cu, Gd, H, K, Li, Mg, Na, Yb, Ba, Ce, Dy, Er, Eu, Hg, Ho, In, Nd, Pb, Pr, Rb, Sc, Sm, Sr, Tb, Tl, Tm,	34	Forms “pseudo-alloy”, not considered further

**Table 3:** Optimized parameters for the polynomial model for $\tau^{\prime }$ and $E_{int}^{0}$.

	$a_{1}$	$a_{2}$	$a_{3}$	$a_{4}$	$a_{5}$
$\tau^{\prime }$	0.00862105	0.0009171	−0.33872123	0.01492073	−0.10378509
$E_{int}^{0}$ (eV)	0.00987929	−0.0583789	−0.20945502	−0.01081567	−0.00574453

**Table 4:** TW model parameters for 28 alloying elements.

Element X	$\tau^{\prime }$		$E_{int}^{0}$(eV)	
	From Model	From DFT	From Model	From DFT
Hf	0.48	0.49	0.12	0.09
Ta	0.21	0.44	0.15	0.13
Re	−0.42	−0.84	−0.27	−0.15
Os	−0.68	−1.78	−0.60	−0.52
Ir	−0.83	−1.04	−0.93	−0.84
Pt	−0.83	−0.12	−1.19	−0.88
Si	1.11	/	−0.20	−0.448
W	0.10 (−0.10) *	/	−0.01	/
Ti	0.54	/	0.08	/
V	0.24	/	0.12	/
Cr	0.10 (−0.10) *	/	0.08 (−0.006) *	/
Mn	−0.45	/	−0.25	/
Fe	−0.74	/	−0.56	/
Co	−0.92	/	−0.86	/
Ni	−0.95	/	−1.10	/
Ga	0.42	/	−0.87	/
Ge	1.54	/	−0.27	/
Zr	0.61	/	0.11	/
Nb	0.33	/	0.14	/
Ru	−0.60	/	−0.57	/
Rh	−0.78	/	−0.89	/
Pd	−0.79	/	−1.14	/
B	−0.62	/	−0.76	/
C	0.47	/	−0.15	/
N	1.97	/	0.83	/
Al	0.01	/	−0.81	/
P	2.63	/	0.76	/
Lu	0.64	/	−0.14	/

* Values in brackets are from the polynomial model, but adjusted manually to the unbracketed value based on experimental information on hardness-composition relationship [36].

## Data Availability

The datasets presented in this article are not readily available because the data are part of an ongoing study.

## Supplementary Materials

The following supporting information can be downloaded at: https://www.mdpi.com/article/10.3390/ma18143329/s1, Calculated Mo-X binary phase diagrams.
