# Supplementary material for: Evaluating Binary Molybdenum Alloys as Strong and Ductile High-Temperature Materials
Source: Materials (Basel). 2025 Jul 15;18(14):3329. doi: 10.3390/ma18143329 (PMC12299779; doi:10.3390/ma18143329)
Supplement: Supplementary file 1 [file materials-18-03329-s001.zip › materials-3711209-supplementary.pdf]

Supplementary Material to “Evaluating binary molybdenum alloys as strong and ductile high-temperature materials” by J. Yan, C. Fu et al.

Calculated Mo–X Binary Phase Diagrams (Types I, II, III)

Mo-Ti (TCNI10)

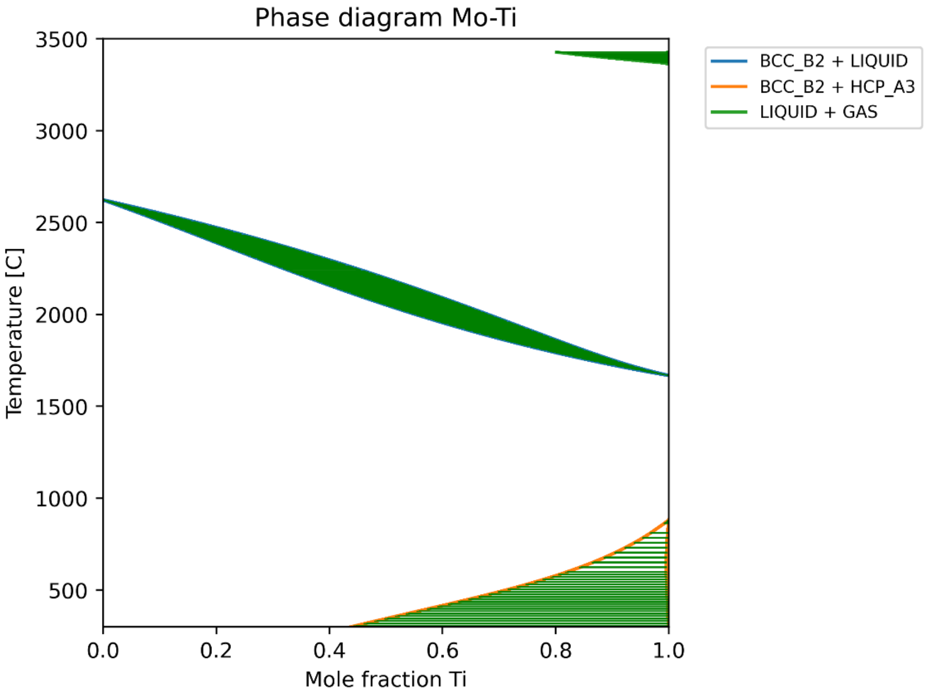

Mo-V (TCNI10)

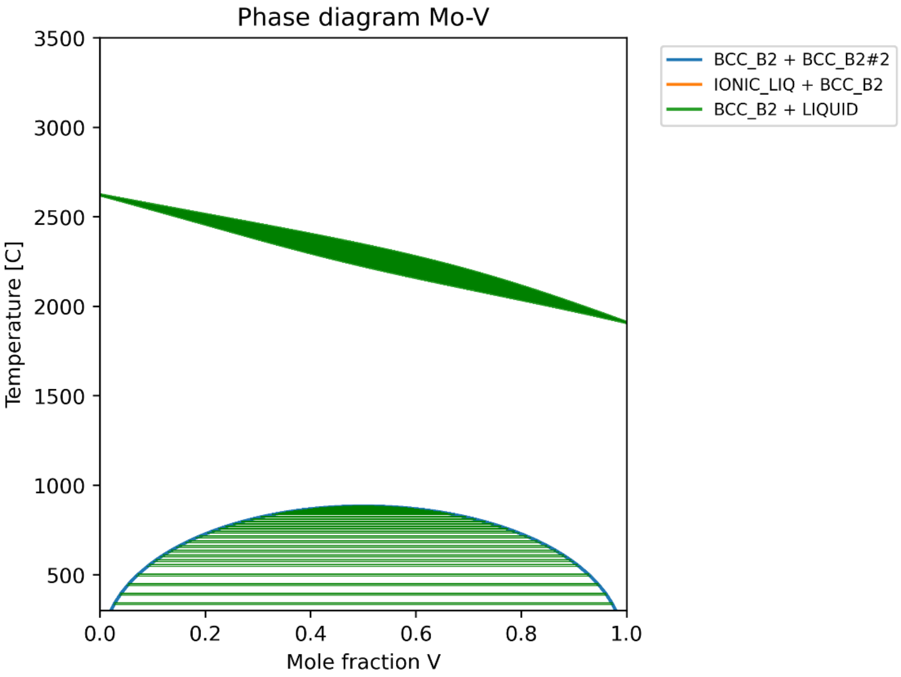

Mo-W (TCNI10)

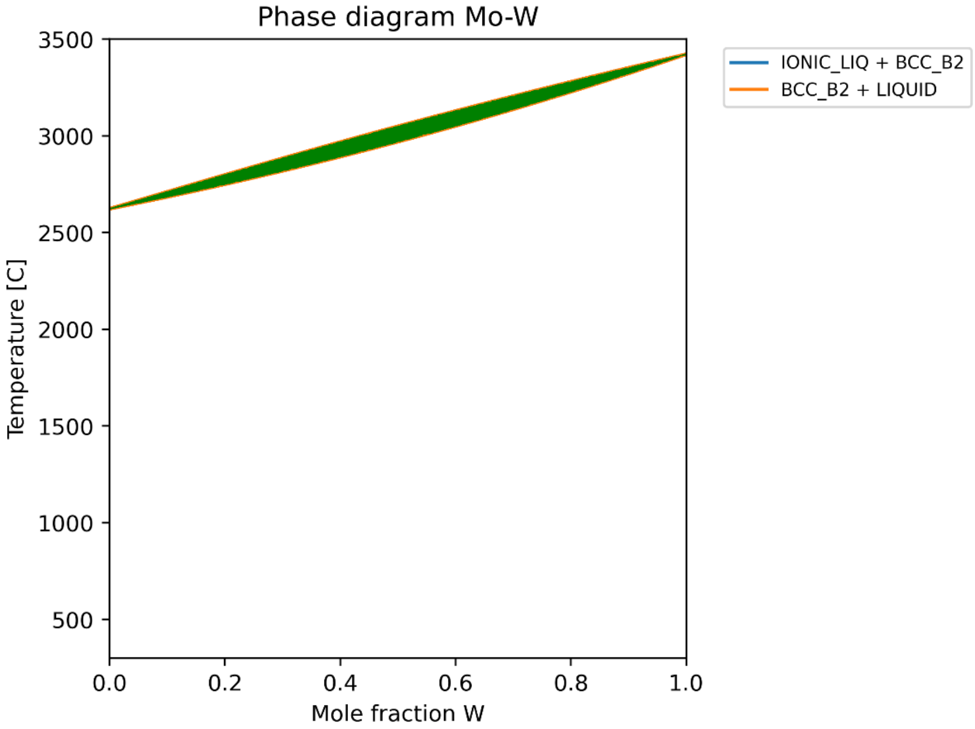

Mo-Nb (TCNI10)

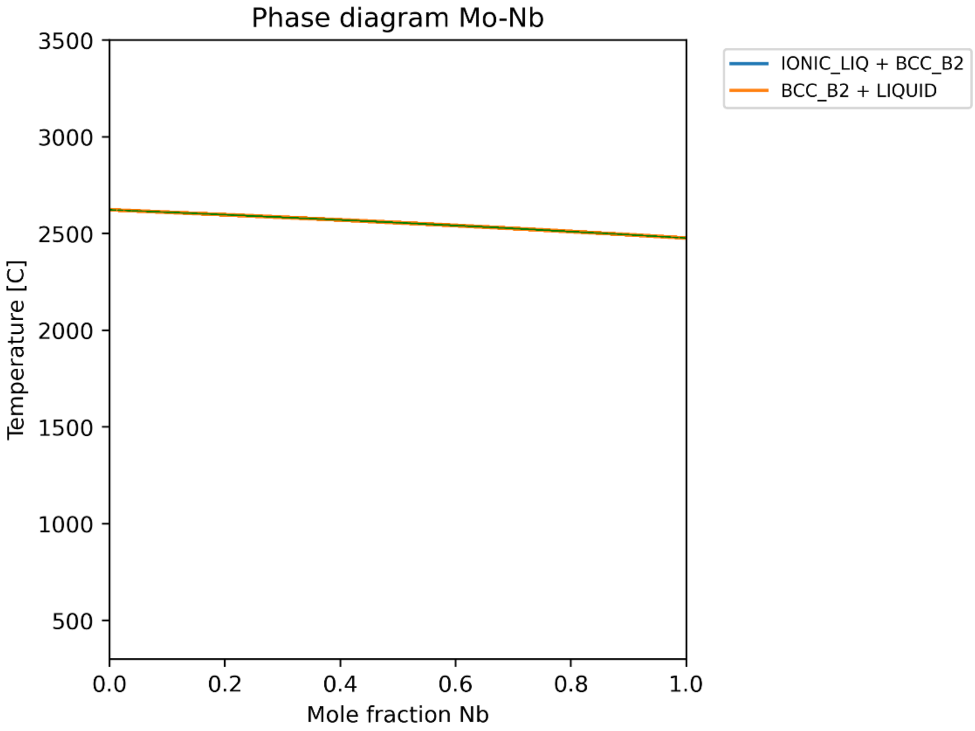

Mo-Cr (TCNI10)

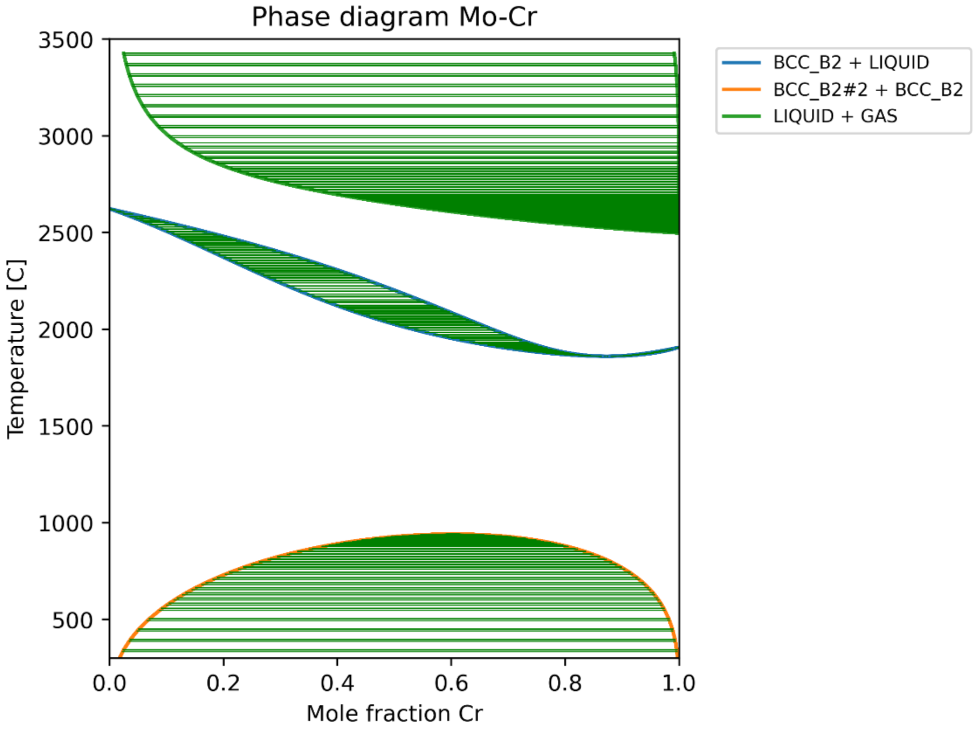

Mo-Ta (TCNI10)

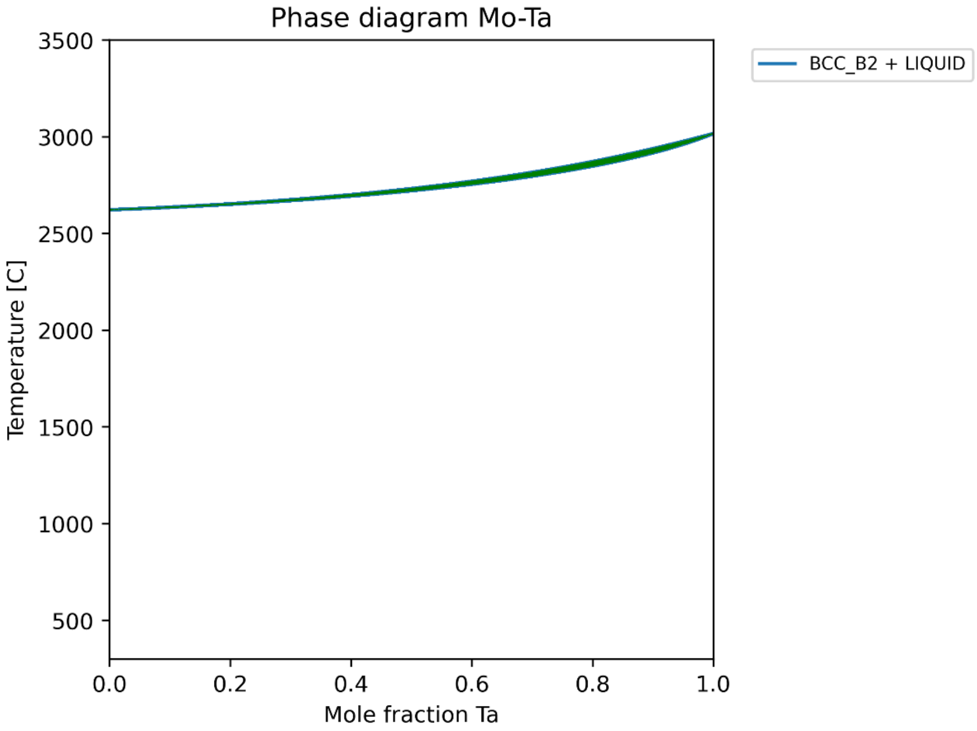

Mo-Al (TCNI10)

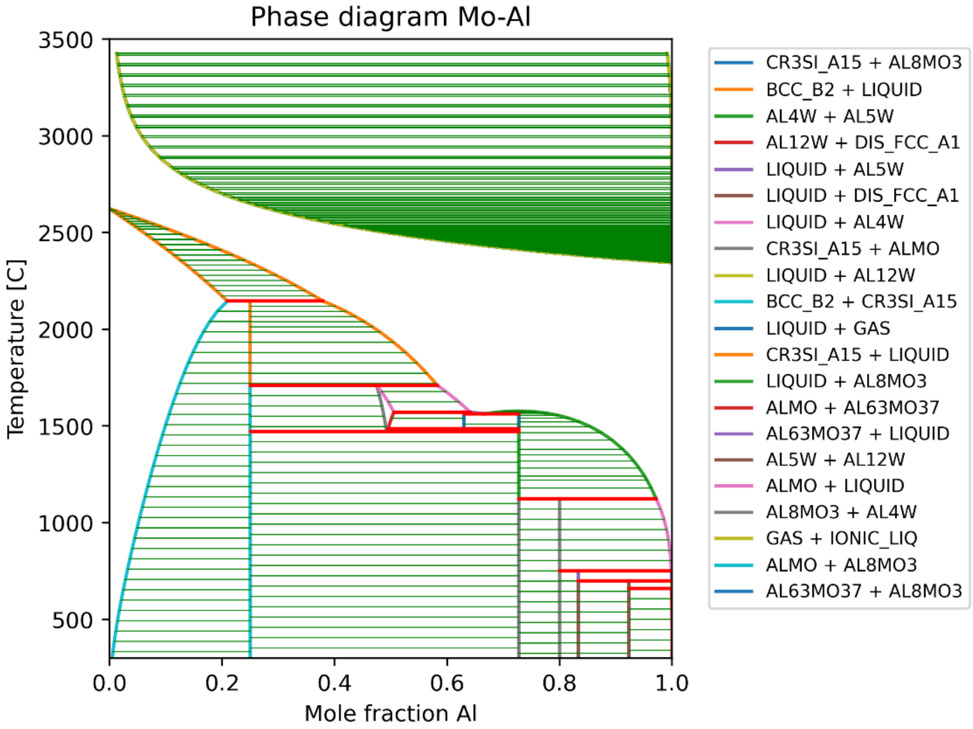

Mo-B (TCNI10)

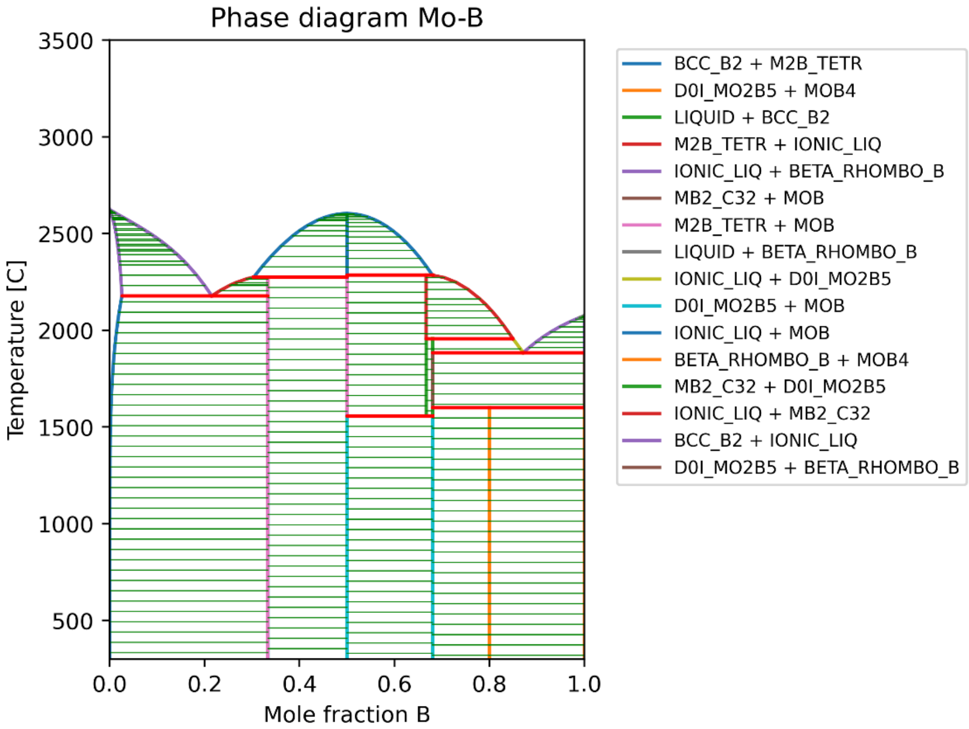

Mo-C (TCNI10)

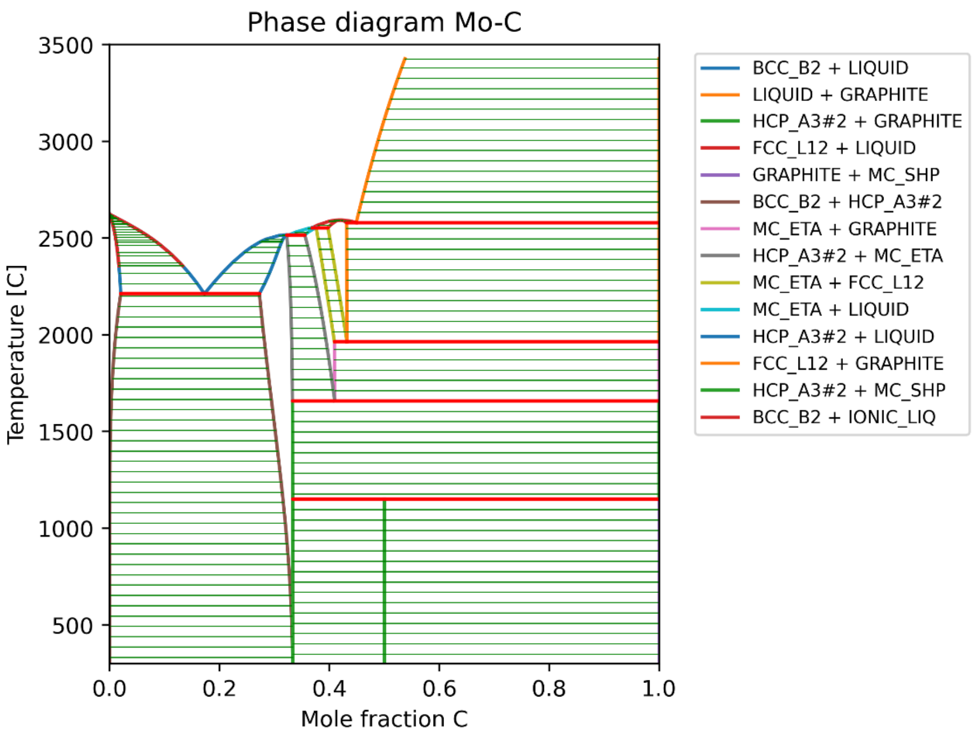

Mo-Co (TCNI10)

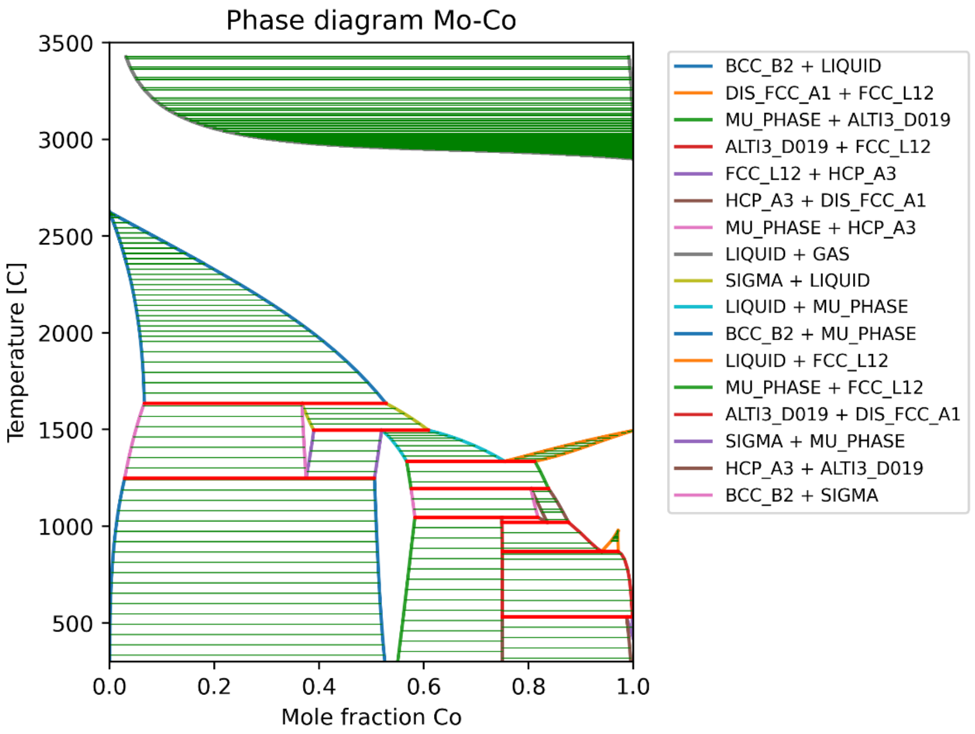

Mo-Fe (TCNI10)

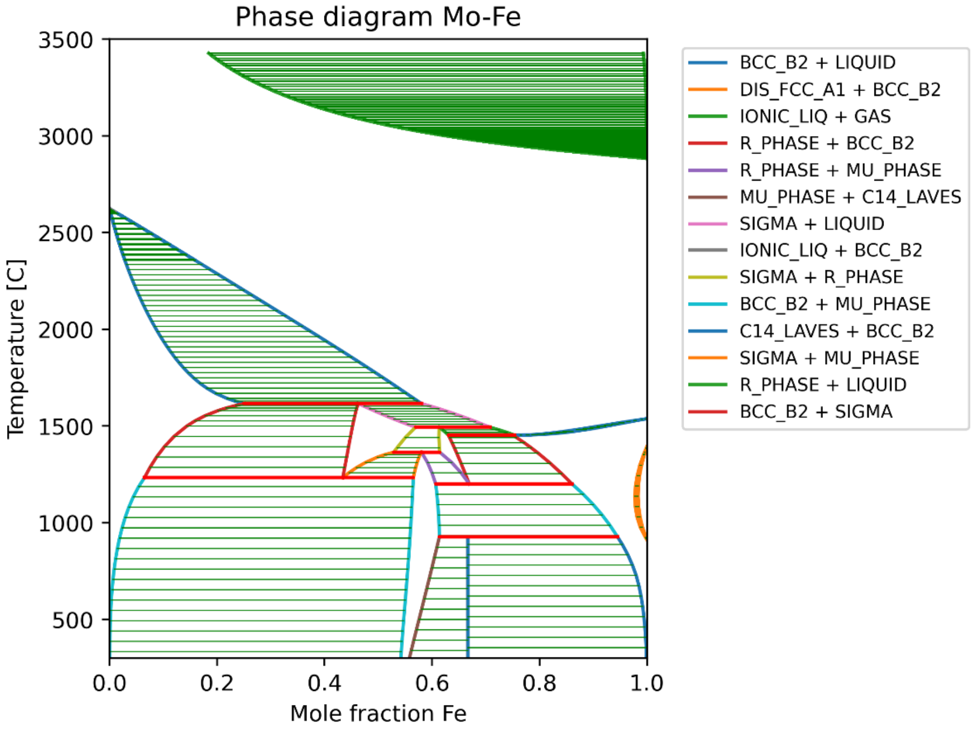

Mo-Ga (Landolt-Börnstein handbook) (omitted)

Mo-Ge (Landolt-Börnstein handbook) (omitted)

Mo-Hf (TCNI10)

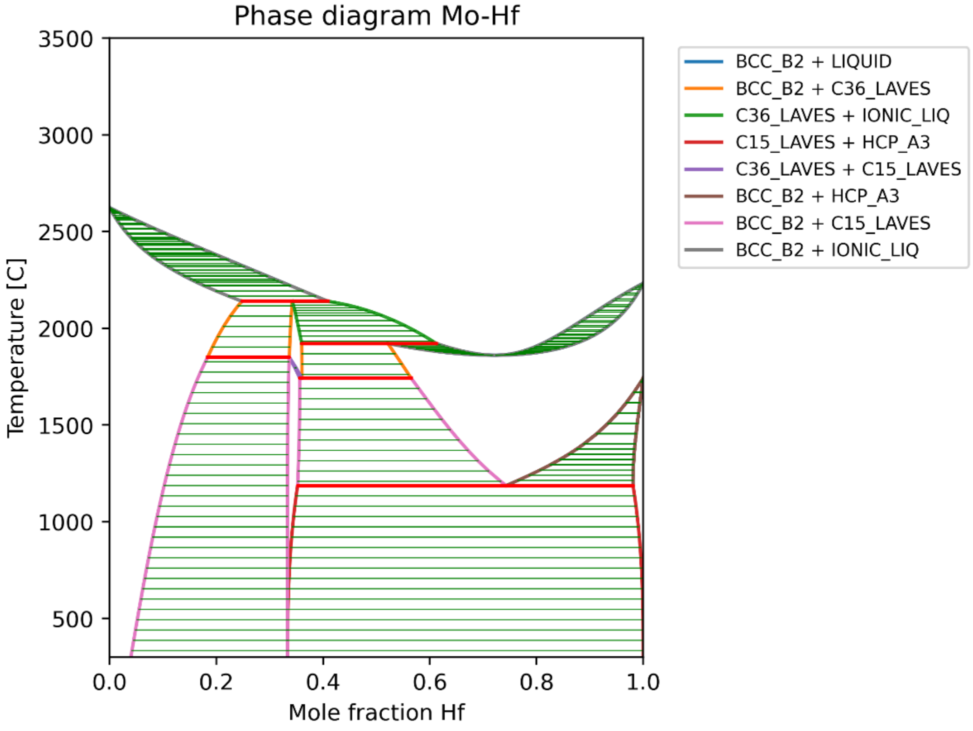

Mo-Ir (TCHEA6)

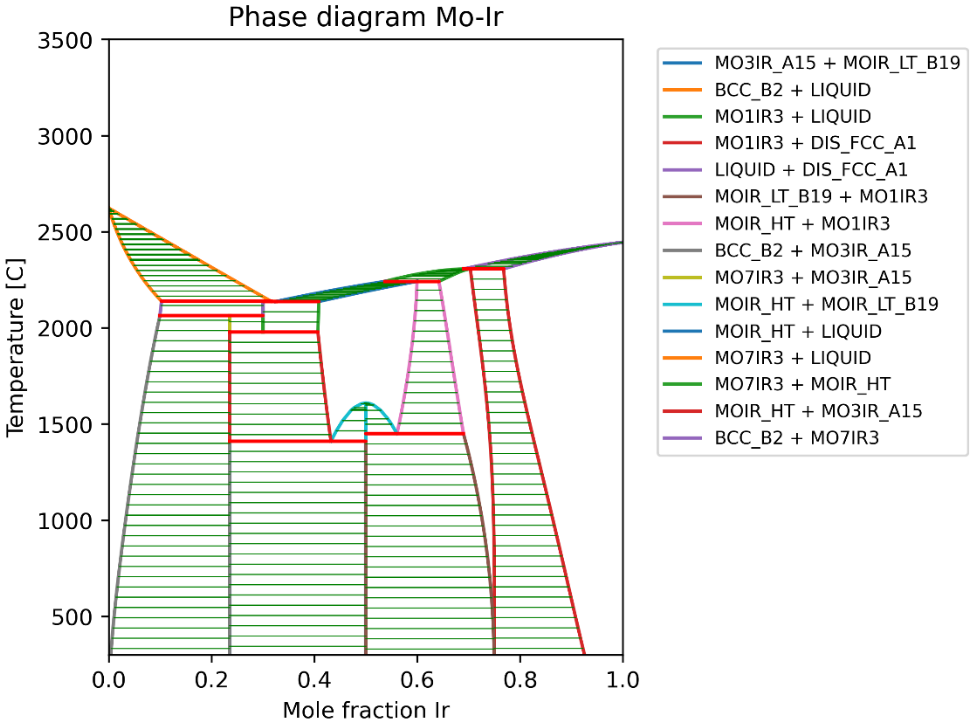

Mo-Mn (TCNI10)

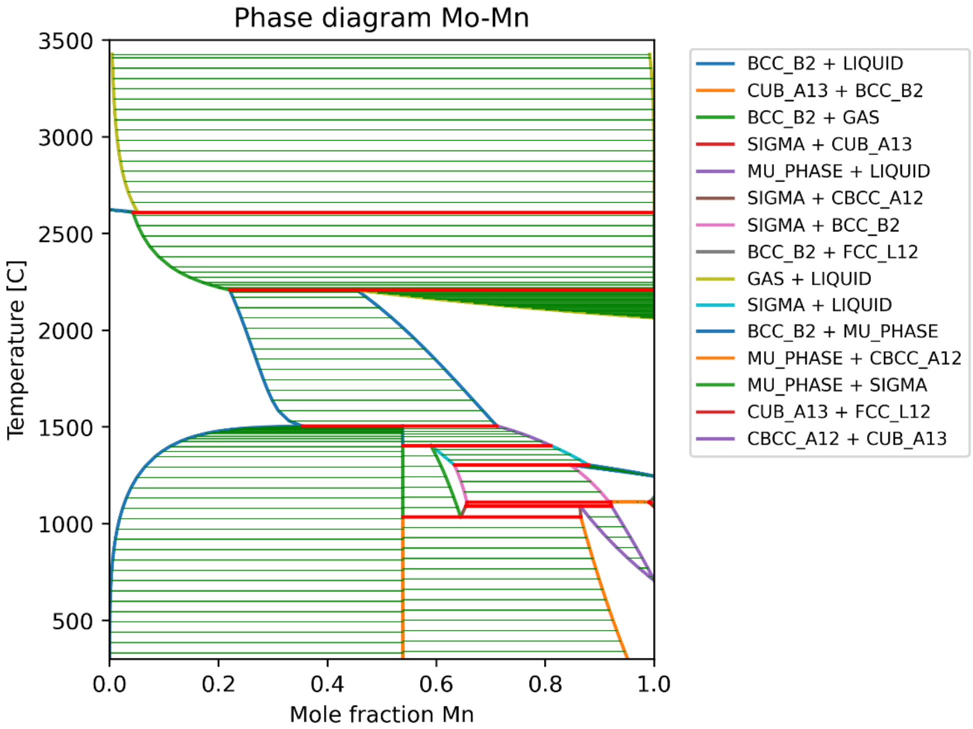

Mo-N (TCNI10，P=10^5Pa)

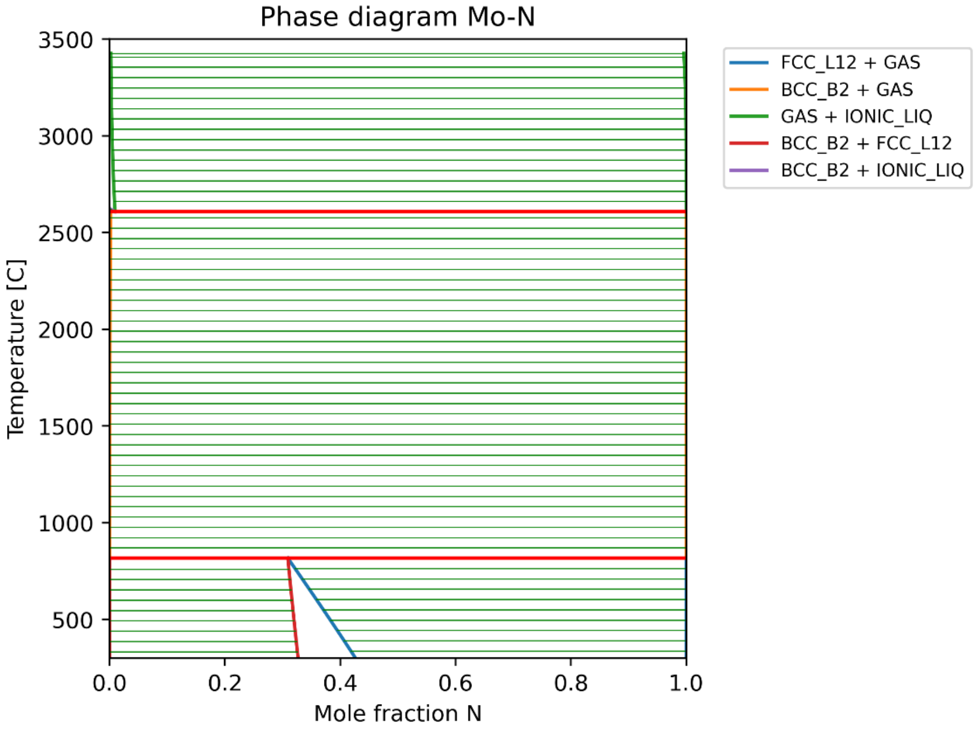

Mo-Ni (TCNI10)

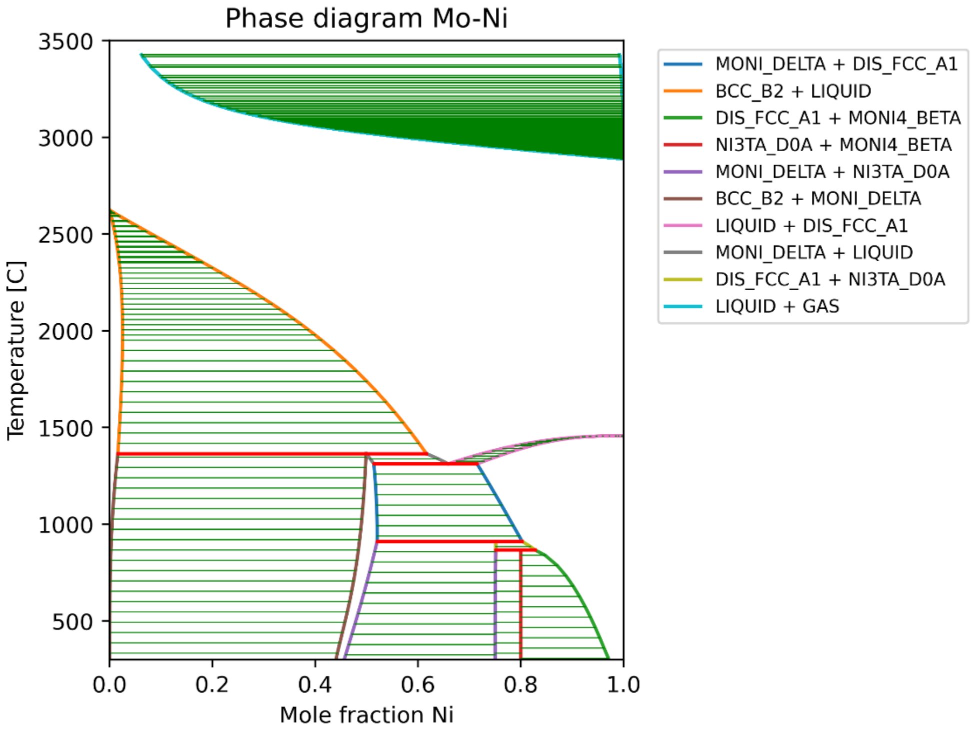

Mo-Os (Landolt-Börnstein handbook) (omitted)

Mo-P (TCFE10)

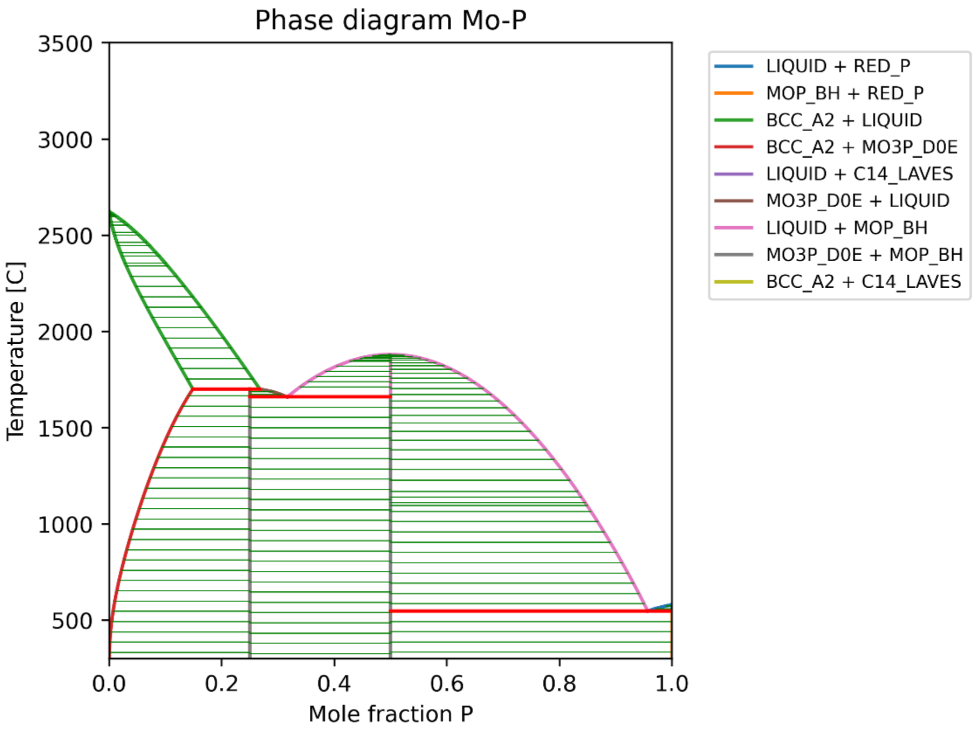

Mo-Pd (TCNI10)

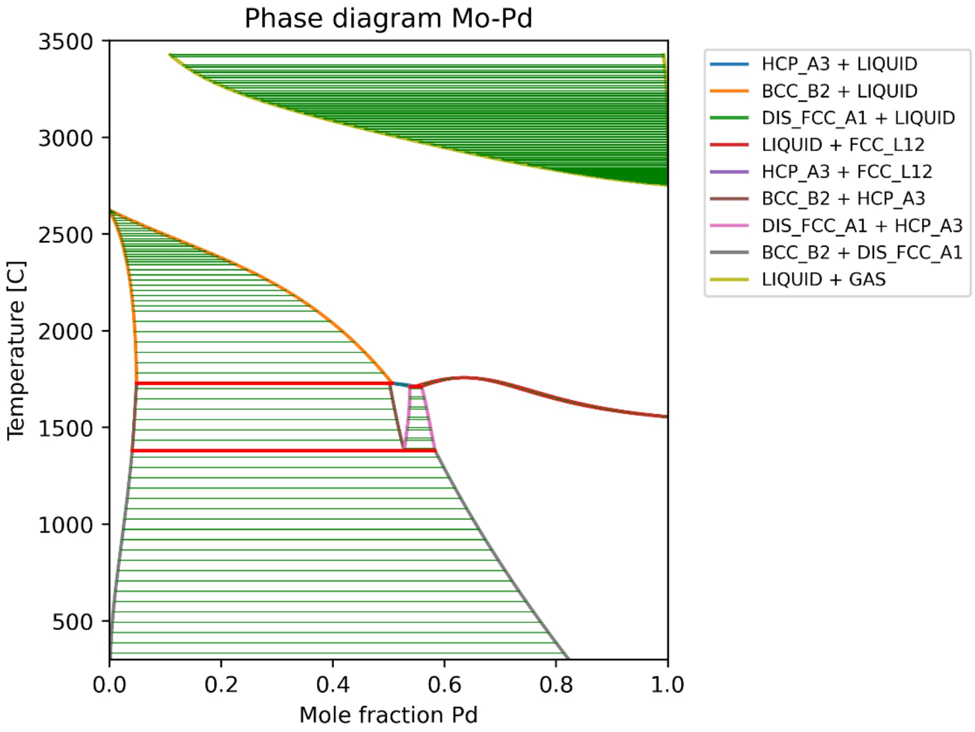

Mo-Pt (TCNI10)

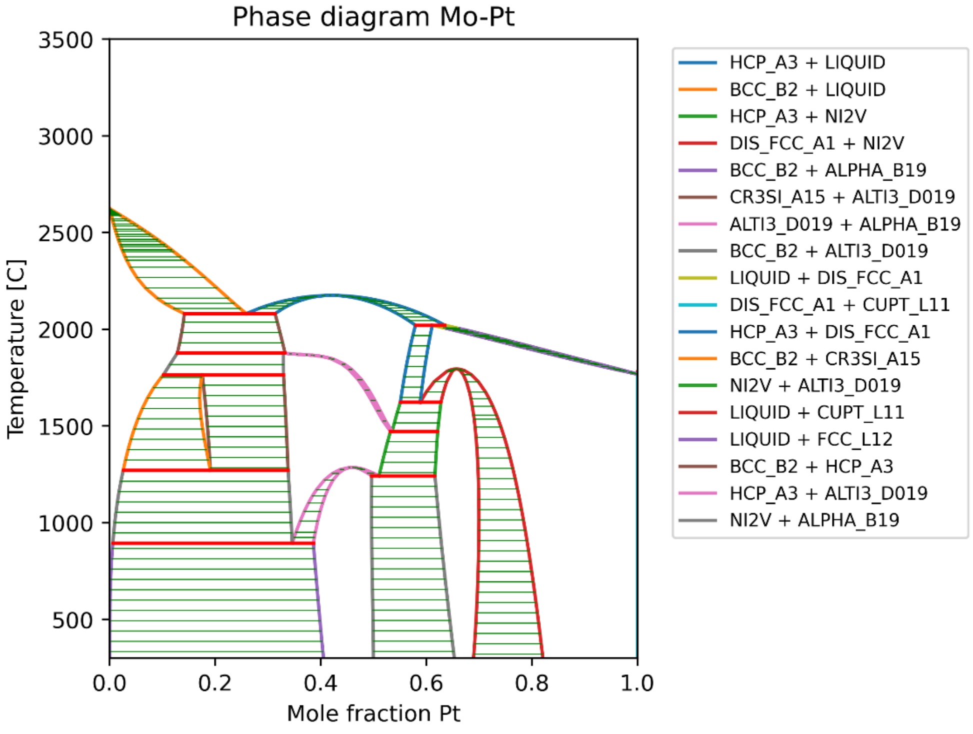

Mo-Re (TCNI10)

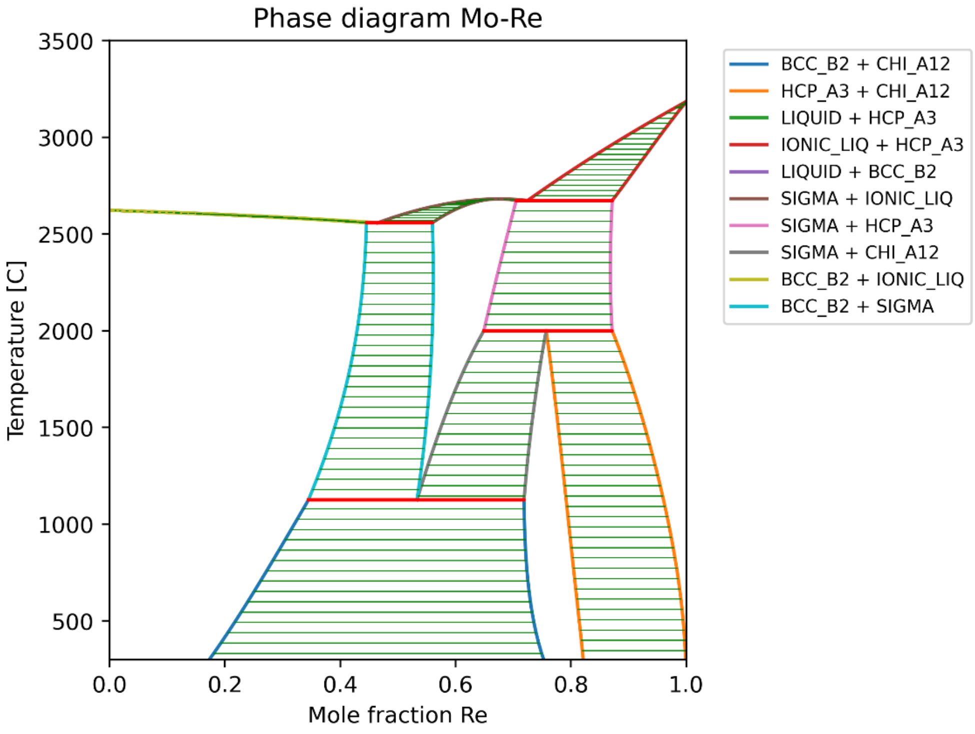

Mo-Rh (TCHEA6)

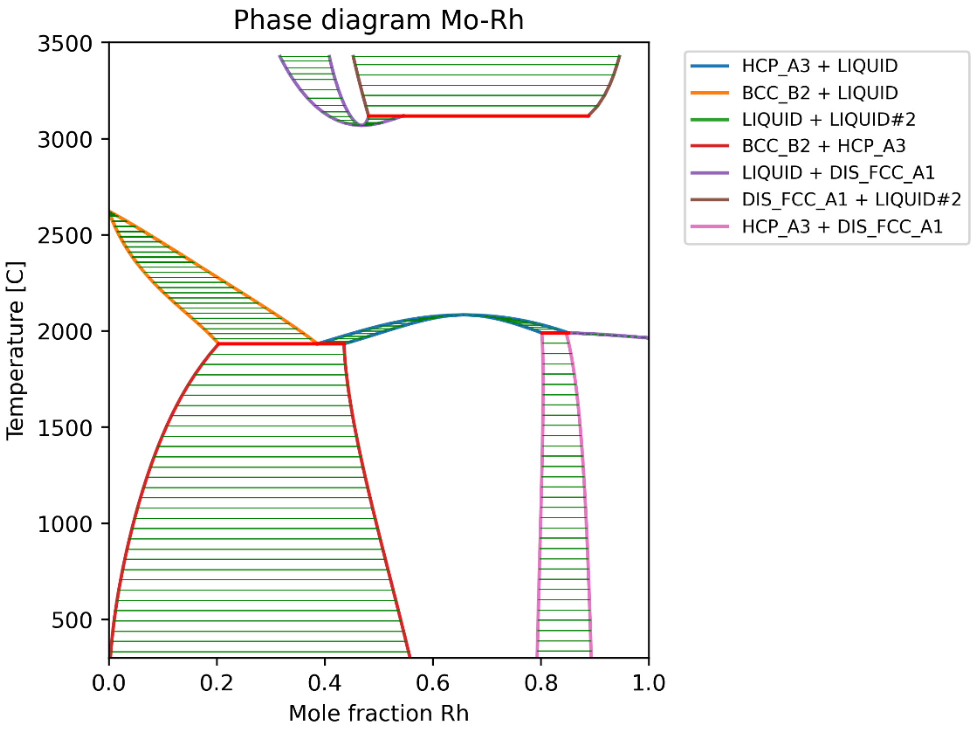

Mo-Ru (TCNI10)

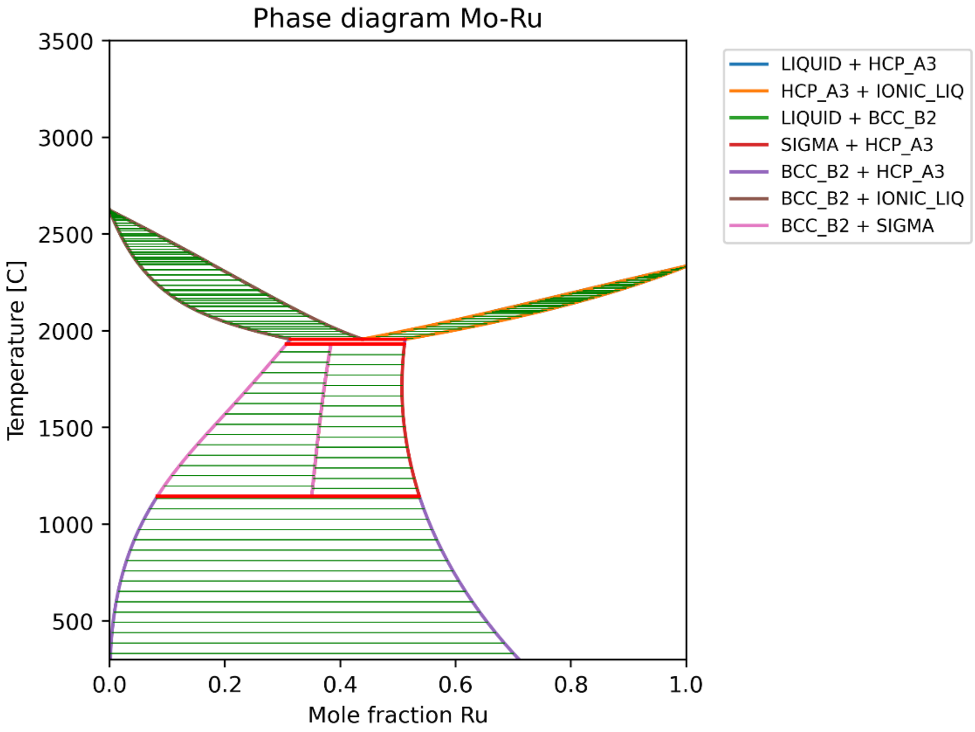

Mo-Si (TCNI10)

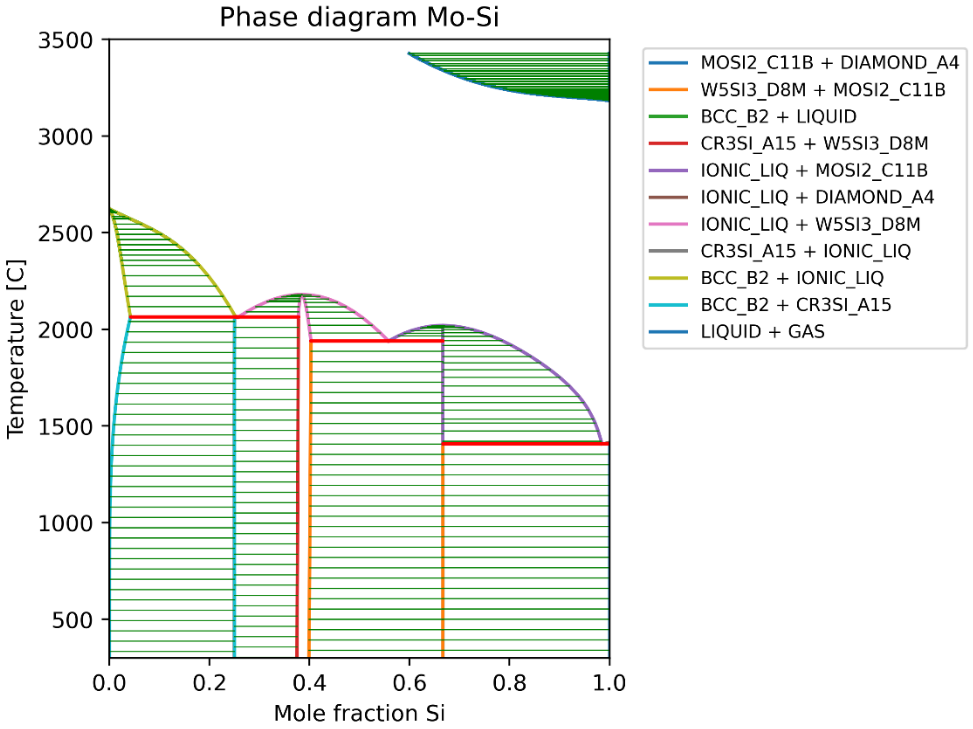

Mo-Zr (TCNI10)

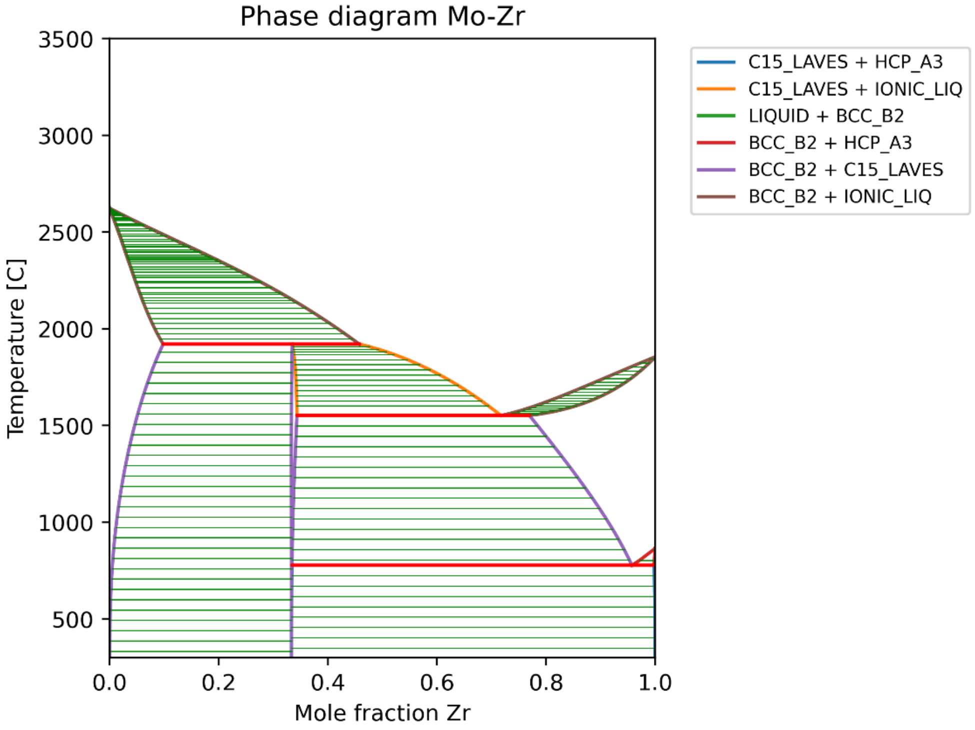

Mo-Lu (Landolt-Börnstein handbook) (omitted)
